# Transcriptome sequencing of *Mycosphaerella fijiensis* during association with *Musa acuminata* reveals candidate pathogenicity genes

**DOI:** 10.1186/s12864-016-3031-5

**Published:** 2016-08-30

**Authors:** Roslyn D. Noar, Margaret E. Daub

**Affiliations:** 1 Department of Plant Pathology, North Carolina State University, Raleigh, NC 27695-7616 USA; 2 Department of Plant and Microbial Biology, North Carolina State University, Raleigh, NC 27695-7612 USA

**Keywords:** *Mycosphaerella fijiensis*, Black Sigatoka, Transcriptome, Effectors, Secondary metabolism, Non-ribosomal peptide synthase, Fusicoccane, Domain of Unknown Function 3328, Salicylate hydroxylase, Dispensable chromosome

## Abstract

**Background:**

*Mycosphaerella fijiensis*, causative agent of the black Sigatoka disease of banana, is considered the most economically damaging banana disease. Despite its importance, the genetics of pathogenicity are poorly understood. Previous studies have characterized polyketide pathways with possible roles in pathogenicity. To identify additional candidate pathogenicity genes, we compared the transcriptome of this fungus during the necrotrophic phase of infection with that during saprophytic growth in medium.

**Results:**

Transcriptome analysis was conducted, and the functions of differentially expressed genes were predicted by identifying conserved domains, Gene Ontology (GO) annotation and GO enrichment analysis, Carbohydrate-Active EnZymes (CAZy) annotation, and identification of genes encoding effector-like proteins. The analysis showed that genes commonly involved in secondary metabolism have higher expression in infected leaf tissue, including genes encoding cytochrome P450s, short-chain dehydrogenases, and oxidoreductases in the 2-oxoglutarate and Fe(II)-dependent oxygenase superfamily. Other pathogenicity-related genes with higher expression in infected leaf tissue include genes encoding salicylate hydroxylase-like proteins, hydrophobic surface binding proteins, CFEM domain-containing proteins, and genes encoding secreted cysteine-rich proteins characteristic of effectors. More genes encoding amino acid transporters, oligopeptide transporters, peptidases, proteases, proteinases, sugar transporters, and proteins containing Domain of Unknown Function (DUF) 3328 had higher expression in infected leaf tissue, while more genes encoding inhibitors of peptidases and proteinases had higher expression in medium. Sixteen gene clusters with higher expression in leaf tissue were identified including clusters for the synthesis of a non-ribosomal peptide. A cluster encoding a novel fusicoccane was also identified. Two putative dispensable scaffolds were identified with a large proportion of genes with higher expression in infected leaf tissue, suggesting that they may play a role in pathogenicity. For two other scaffolds, no transcripts were detected in either condition, and PCR assays support the hypothesis that at least one of these scaffolds corresponds to a dispensable chromosome that is not required for survival or pathogenicity.

**Conclusions:**

Our study revealed major changes in the transcriptome of *Mycosphaerella fijiensis*, when associating with its host compared to during saprophytic growth in medium. This analysis identified putative pathogenicity genes and also provides support for the existence of dispensable chromosomes in this fungus.

**Electronic supplementary material:**

The online version of this article (doi:10.1186/s12864-016-3031-5) contains supplementary material, which is available to authorized users.

## Background

Banana (including common “dessert” bananas as well as cooking and plantain types) is one of the world’s most important food crops, grown in tropical and subtropical regions in over 120 countries [1]. Only about 10–15 % of bananas are grown for export, with the rest serving as an important subsistence crop in many developing countries [2, 3]. Black Sigatoka, caused by the ascomycete fungus *Mycosphaerella fijiensis*, is a major threat to banana production. It is found in almost all banana-growing countries, and can cause up to 50 % yield loss as well as premature ripening of fruit [3]. Control of the disease is through frequent applications of fungicides, which are estimated to account for 25–30 % of the total banana production cost [1–4]. Fungicide resistance is an ongoing problem that threatens the viability of this method of black Sigatoka control [2, 5].

*M. fijiensis* produces both conidia and ascospores, both of which can infect banana leaves via the stomata [1]. The fungus is a hemibiotroph. The conidia or ascospores germinate, forming mycelium that initially grows epiphytically on the leaf surface prior to penetration through the stomata and into the leaf [1]. The fungus colonizes the intercellular spaces of the leaf during its biotrophic phase [1]. The fungus then switches to a necrotrophic stage, leading to the death of leaf cells and the formation of necrotic leaf lesions [1].

As a hemibiotroph, *M. fijiensis* would be expected to produce both effectors to suppress host defense responses and prevent death of host cells during biotrophy [6], and toxic secondary metabolites and proteins to kill host tissue during necrotrophy [7]. However, little is known about the repertoire of effectors and toxic metabolites produced by *M. fijiensis* during its association with banana. Homologs of the *Cladosporium fulvum* effectors Ecp2, Ecp6, and Avr4 have been identified from the *M. fijiensis* genome [8, 9]. However, most fungal pathogens have a large repertoire of effectors, and most effectors have a restricted phylogenetic distribution, so *M. fijiensis* is likely to produce many other effectors [6]. In addition to effectors, several studies have been done to identify toxins secreted by *M. fijiensis*. Several phytotoxic metabolites have been identified from *M. fijiensis* including 2,4,8-trihydroxytetralone, which showed some host selectivity and was thought to be an important pathogenicity factor [10–12]. However, 2,4,8-trihydroxytetralone is a melanin shunt metabolite [13], and disruption of the melanin biosynthetic pathway was shown to have no effect on pathogenicity [1]. Phytotoxic activity has also been identified from the hydrophilic portion of culture filtrates, but the identity of these toxins is unknown [14, 15]. All of these studies were done using mycelium grown in culture conditions, which may not fully reflect what is produced during the association of *M. fijiensis* with its host.

In previous work we used the publicly available *M. fijiensis* genome sequence (NCBI Genome ID 10962) [16], obtained from isolate CIRAD86, to predict the capacity of *M. fijiensis* to produce polyketides [17], an important class of secondary metabolites that are used as pathogenicity factors by closely related fungi [18, 19]. In this study, seven putative polyketide synthase gene clusters and one hybrid polyketide synthase/non-ribosomal peptide synthase gene cluster were identified [17]. Among the clusters were ones with similarity to clusters producing melanin, as well as the secondary metabolites fumonisin, solanapyrone, and alternapyrone produced by *Alternaria* and *Fusarium* species [17]. Melanin has been shown to play important roles in fungal pathogenicity of plants including penetration into host tissue [20, 21]. Fumonisin promotes *Fusarium* spp. pathogenicity by perturbing sphingolipid biosynthesis in the host [22, 23].

The publicly available *M. fijiensis* genome sequence has also been used to investigate possible dispensable chromosomes. Many fungi use genes located on conditionally dispensable chromosomes to assist in pathogenicity, host specificity, and other functions that are useful but not required for survival [24]. Ohm et al. observed that the CIRAD86 *M. fijiensis* genome contains 14 scaffolds that are very different from the rest of the scaffolds in the genome: they are small, have a low G + C content, have the lowest gene density and the lowest proportion of genes encoding proteins with PFAM domains, have the highest proportion of repetitive DNA, and have different codon usage [25]. Though it has not been proven that these 14 scaffolds represent dispensable chromosomes, they share their unusual characteristics with dispensable chromosomes from the related species *Mycosphaerella graminicola* [25, 26].

Next-generation transcriptome sequencing has greatly improved our understanding of the genetic mechanisms of pathogenicity in other species [27, 28]. For *M. fijiensis*, it can help identify genes encoding effectors, secondary metabolite pathways, and other proteins that may be important for pathogenicity. It can also identify portions of the genome with an abundance of genes that are expressed during association with banana, and thereby suggest which putative dispensable chromosomes may be important for pathogenesis. To date, however, research on changes in gene expression during the *M. fijiensis*-*Musa* spp. (banana) interaction has largely been limited to the *Musa* spp. transcriptome. Portal et al. created suppression subtractive hybridization cDNA libraries from late stages of infection to identify expressed genes from banana and *M. fijiensis* [29]. They identified banana genes involved in biosynthesis of phenyl-propanoids, jasmonic acid and ethylene, genes encoding pathogenesis-related (PR) proteins, and genes involved in detoxification such as glutathione S-transferases [29]. Although many defense-related banana genes were identified, the inefficiency of CTAB-based RNA extraction protocols with *M. fijiensis* had not been reported at the time [29]. As a result, the only fungal gene identified from their libraries was a gene for UDP glucose pyrophosphorylase, which is involved in trehalose biosynthesis [29]. Another study used microarray analysis to compare genes expressed in the resistant banana variety Calcutta 4 versus the susceptible variety, Williams, when challenged with *M. fijiensis* [30]. Banana genes encoding peroxidase, PR-4, PR-10, phenylalanine ammonia lyase, and disease resistance response 1 showed higher expression in Calcutta 4 compared to Williams, between 6 and 24 h after inoculation [30].

Studies of *M. fijiensis* gene expression have been more limited. Expressed sequence tags have been identified from *M. fijiensis* grown in three different culture media: Potato Dextrose Agar (PDA), Fries liquid medium, and Fries liquid medium with banana leaf extract added [31]. This analysis found a homolog of the *Cladosporium fulvum* effector gene *Avr4* in all three libraries, and a homolog of the *C. fulvum* effector gene *Ecp2* in both the Fries liquid medium and medium supplemented with leaf extract [31]. In our study on *M. fijiensis* polyketide synthases, we used transcriptome sequencing to compare expression of the polyketide synthase gene clusters in infected banana leaf tissue relative to in culture medium [17]. The genes in the previously mentioned polyketide synthase clusters with similarities to fumonisin and solanapyrone clusters had increased expression in infected leaf tissue, suggesting that these gene clusters may produce polyketide products that are important for pathogenicity [17]. By contrast, the melanin cluster had lower expression in infected leaf tissue as compared to expression in culture medium [17]. This was the first study in which transcriptome sequencing was used to analyze expression of *M. fijiensis* during its association with banana. However, our analysis was limited to polyketide synthase gene clusters.

Other transcriptome sequencing studies have been done with banana infected with the related banana pathogens *Mycosphaerella musicola* and *Mycosphaerella eumusae*. One study compared the resistant banana variety Calcutta 4 to the susceptible Grand Nain variety during association with *M. musicola* [32]. A homolog of the *C. fulvum* effector *Ecp6* gene [8] was shown to have higher expression in *M. musicola* during association with Calcutta 4 relative to Grand Nain, whereas genes encoding a SAP family cell cycle dependent phosphate-associated protein, two Hsp70 family proteins, an FAD binding domain protein, and a calcium channel all had higher expression in *M. musicola* in the association with Grand Nain compared to Calcutta 4 [32]. Another study compared the transcriptomes of resistant (Manoranjitham) and susceptible (Grand Nain) banana varieties challenged or unchallenged with *M. eumusae* [33]. Banana genes with higher expression in the resistant compared to susceptible banana variety included those encoding enzymes involved in the phenylpropanoid pathway, abscisic acid biosynthesis, alkaloid biosynthesis, and scavenging of reactive oxygen species [33].

The limited information on pathogenicity-related genes in *M. fijiensis* and other *Mycosphaerella* banana pathogens is a barrier to the development of new control methods for this devastating disease. Thus, the objectives of this paper were to expand beyond our focus on polyketides to use transcriptome sequencing data from symptomatic leaf tissue and mycelium growing saprophytically in medium to predict other *M. fijiensis* genes that may have roles in pathogenicity. In addition, we were interested to determine if putative pathogenicity-related genes are concentrated in particular regions of the genome, such as on scaffolds predicted by Ohm et al. to represent dispensable chromosomes [25].

## Results

### Identification of differentially expressed genes

Banana plants and Potato Dextrose Broth (PDB) medium were inoculated with *M. fijiensis* conidia. Tissue harvested from the fungus grown in liquid medium as well as from symptomatic leaf tissue were used for RNA isolation and transcriptome sequencing. Principal component analysis showed that the samples from the same treatment (leaf tissue, liquid medium) clustered together, separately from samples in the other treatment group (Additional file 1: Figure S1). A total of 802 differentially expressed genes were identified (Additional file 2: Figure S2, Additional file 3: Tables S1 and Additional file 4: Table S2); of these, 483 genes were more highly expressed in infected leaf tissue, and 319 genes were more highly expressed in culture medium (Additional file 4: Table S2).

To identify the genes with the greatest differential expression, differentially expressed genes were sorted based on their log_2_ fold change (log2FC) values. Lists of the most differentially expressed genes (the 20 with highest and 20 with lowest expression in infected leaf tissue compared to culture medium) can be seen in Tables 1 and 2, respectively. The majority of the genes (13 of the top 20) with higher expression in infected leaf tissue are predicted to encode hypothetical proteins, 11 of which have no conserved domains and two of which have a DUF (Domain of Unknown Function) 3328 domain (Table 1). Other than genes encoding hypothetical proteins, two of the genes most highly expressed in the banana leaf relative to culture medium encode proteins in a polyketide synthase gene cluster (*PKS7-1*) previously described [17]. Other types of genes with higher expression in infected leaf tissue include those encoding an oxidoreductase, a 2-oxoglutarate and Fe(II)-dependent oxygenase superfamily enzyme, a Major Facilitator Superfamily (MFS) multidrug transporter-like protein, a peptidase, and a transcription factor (Table 1). Of genes with lower expression in infected leaf tissue, a majority (11 out of 20) were also predicted to encode hypothetical proteins (Table 2). One of these hypothetical proteins contains a CFEM (Common in Fungal Extracellular Membrane) domain, a cysteine-rich domain present in some proteins that play important roles in pathogenicity [34]. Genes encoding a cupredoxin, a copper transporter, a cysteine synthase, a 2-oxoglutarate and Fe(II)-dependent oxygenase superfamily enzyme, a cytochrome P450, a heme peroxidase, an α/β-hydroxylase, an oxidoreductase, and a glutamine amidotransferase were also among the top 20 differentially expressed genes with reduced expression in infected leaf tissue (Table 2).

**Table 1:** Genes with highest expression in infected leaf tissue compared to culture medium as determined by log2FC

Gene ID	Protein ID	Predicted function of encoded protein	Log2FC
fgenesh1_kg.9_#_12_#_4417424:1	183842	Hypothetical; 44 % identity to WI-1 adhesin from *Blastomyces dermatitidis*	13.3
estExt_Genewise1Plus.C_90054	157089	Hypothetical with Domain of Unknown Function (DUF) 3328	11.4
Mycfi1.estExt_fgenesh1_pg.C_120010	87989	Hypothetical, no conserved domains	11.3
Mycfi1.fgenesh1_pg.C_scaffold_29000110	84397	Hypothetical, no conserved domains	11.2
Mycfi1.e_gw1.43.35.1	46458	FAD-dependent oxidoreductase	11.1
e_gw1.2.477.1	132918	2-oxoglutarate and Fe(II)-dependent oxygenase superfamily	10.9
fgenesh1_pm.1_#_1217	185508	MFS multidrug transporter-like protein	10.8
fgenesh1_pg.3_#_601	195588	Hypothetical, no conserved domains	10.6
Genemark.4551_g	173539	Hypothetical, no conserved domains	10.6
Mycfi1.gw1.34.54.1	20039	PKS7-1	10.5
fgenesh1_pm.4_#_231	188143	Zinc peptidase	10.5
estExt_Genemark.C_30183	207097	Hypothetical, no conserved domains	10.3
fgenesh1_pm.7_#_421	190048	Monooxygenase in PKS7-1 cluster	10.2
fgenesh1_pg.7_#_244	198484	Hypothetical, no conserved domains	10.0
Genemark.2108_g	171096	Hypothetical, no conserved domains	9.96
Genemark.4552_g	173540	Hypothetical, no conserved domains	9.82
fgenesh1_pg.9_#_17	199506	Hypothetical with DUF3328	9.76
Mycfi1.fgenesh1_pm.C_scaffold_23000030	65382	Hypothetical protein in cupin superfamily	9.69
Mycfi1.e_gw1.22.234.1	43729	Transcription factor	9.55
Mycfi1.fgenesh1_pg.C_scaffold_1001762	76887	Hypothetical, no conserved domains	9.37

Table indicates the JGI gene and protein IDs, the predicted function of the encoded protein based on blast and conserved domains, and the log2FC value

**Table 2:** Genes with lowest expression in infected leaf tissue compared to culture medium as determined by log2FC

Gene ID	Protein ID	Predicted function	Log2FC
fgenesh1_pm.2_#_327	186279	Hypothetical, no conserved domains	−7.1
estExt_Genewise1Plus.C_90903	157466	Cysteine synthase	−6.8
estExt_fgenesh1_kg.C_20418	210593	Hypothetical, no conserved domains	−6.8
gw1.2.3679.1	120453	Heme peroxidase	−6.7
estExt_fgenesh1_pm.C_60071	204084	Hypothetical, no conserved domains	−6.7
fgenesh1_kg.1_#_500_#_4410656:1	181367	Hypothetical, with some homology to phosphate carrier protein	−6.7
fgenesh1_pg.1_#_1546	193207	Hypothetical, no conserved domains	−6.4
fgenesh1_pm.2_#_99	186051	α/β-hydroxylase	−6.4
estExt_fgenesh1_pg.C_80340	216449	Hypothetical, with CFEM domain	−6.3
Mycfi1.e_gw1.1.178.1	26018	Cytochrome P450	−6.2
Mycfi1.estExt_fgenesh1_pg.C_160286	88531	Hypothetical, no conserved domains	−6.2
Mycfi1.e_gw1.3.802.1	31919	FAD dependent oxidoreductase	−6.1
Genemark.2479_g	171467	Hypothetical, no conserved domains	−6.1
estExt_Genemark.C_80180	208708	2-oxoglutarate and Fe(II)-dependent oxygenase superfamily	−6.1
estExt_fgenesh1_kg.C_80203	212291	Glutamine amidotransferase	−6.0
estExt_fgenesh1_kg.C_30187	210875	Copper transporter	−5.9
estExt_fgenesh1_kg.C_50238	211639	Hypothetical, no conserved domains	−5.8
estExt_fgenesh1_kg.C_20200	210380	Hypothetical, no conserved domains	−5.8
estExt_fgenesh1_kg.C_20513	210685	Hypothetical, no conserved domains	−5.8
Mycfi1.estExt_Genewise1.C_12242	48579	Cupredoxin	−5.8

Table indicates the JGI gene and protein IDs, the predicted function of the encoded protein based on blast and conserved domains, and the log2FC value

### Validation of RNA-Seq results by RT-qPCR

**Fig. 1:**
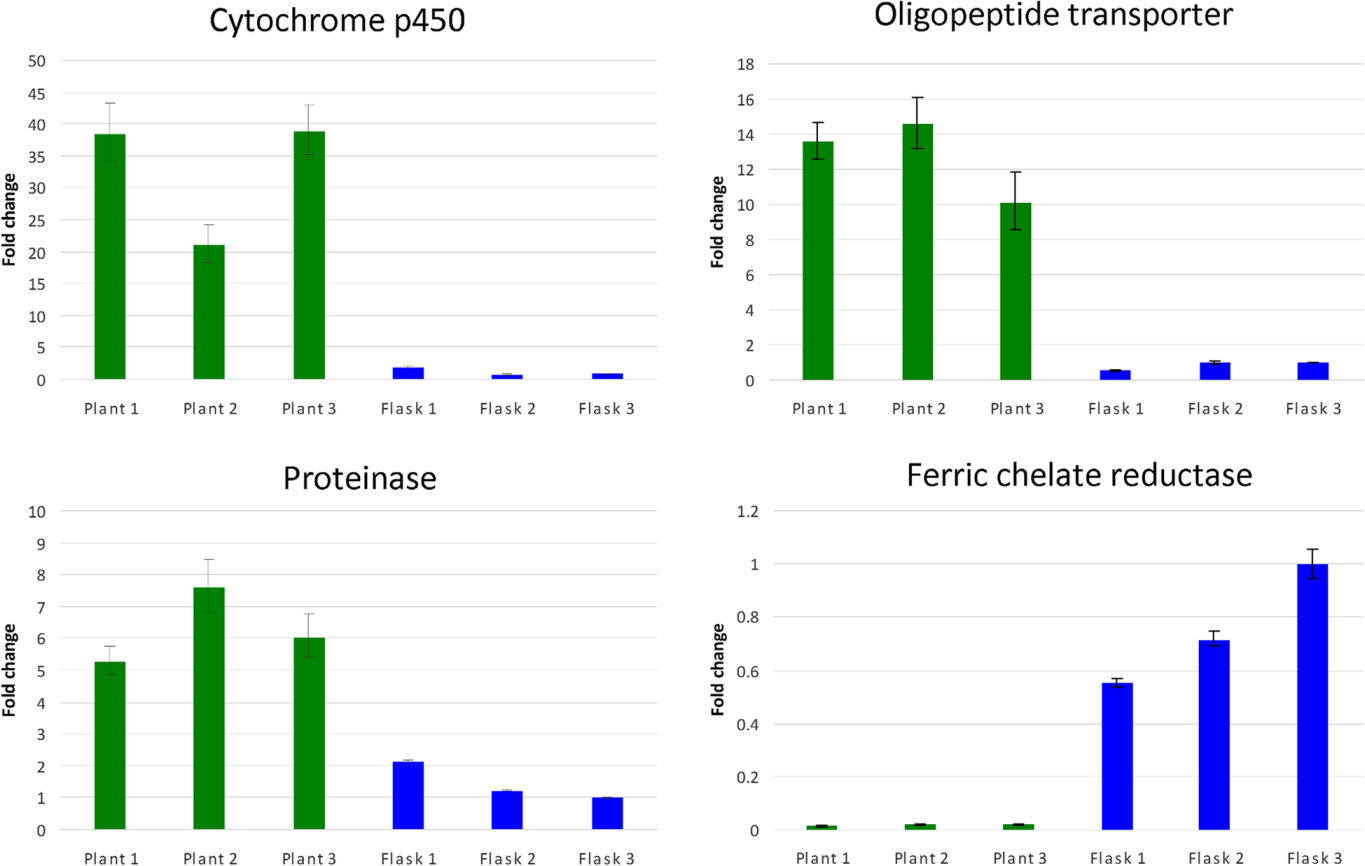
Validation of RNA-Seq results using RT-qPCR assays. The cytochrome P450 and oligopeptide transporter were normalized against ubiquitin-conjugating enzyme E2 6, and the proteinase and ferric-chelate reductase were normalized against transcription initiation factor TFIID subunit 10. Fold change was calculated using the 2^-ΔΔC^_T_ method, relative to the Flask #3 sample. Green bars represent expression in infected leaf tissue; blue bars represent expression in mycelial culture. Error bars represent standard error from mean of technical replicates for each sample. Results confirmed the RNA-Seq data that showed higher expression of the cytochrome P450, proteinase, and oligopeptide transporter in infected leaf tissue, whereas the ferric-chelate reductase had lower expression

To validate the identification of differentially expressed genes from the RNA-Seq dataset, four genes were chosen for further expression analysis using RT-qPCR on the same RNA samples. These genes were: a cytochrome P450, a proteinase, an oligopeptide transporter, and a ferric-chelate reductase. RT-qPCR assays confirmed the RNA-Seq analysis: the cytochrome P450, proteinase, and oligopeptide transporter had higher expression in infected leaf tissue, and the ferric-chelate reductase had lower expression in infected leaf tissue compared to culture medium (Fig. 1).

### Prediction of differentially expressed gene functions

#### Blast and conserved domain analysis

**Fig. 2:**
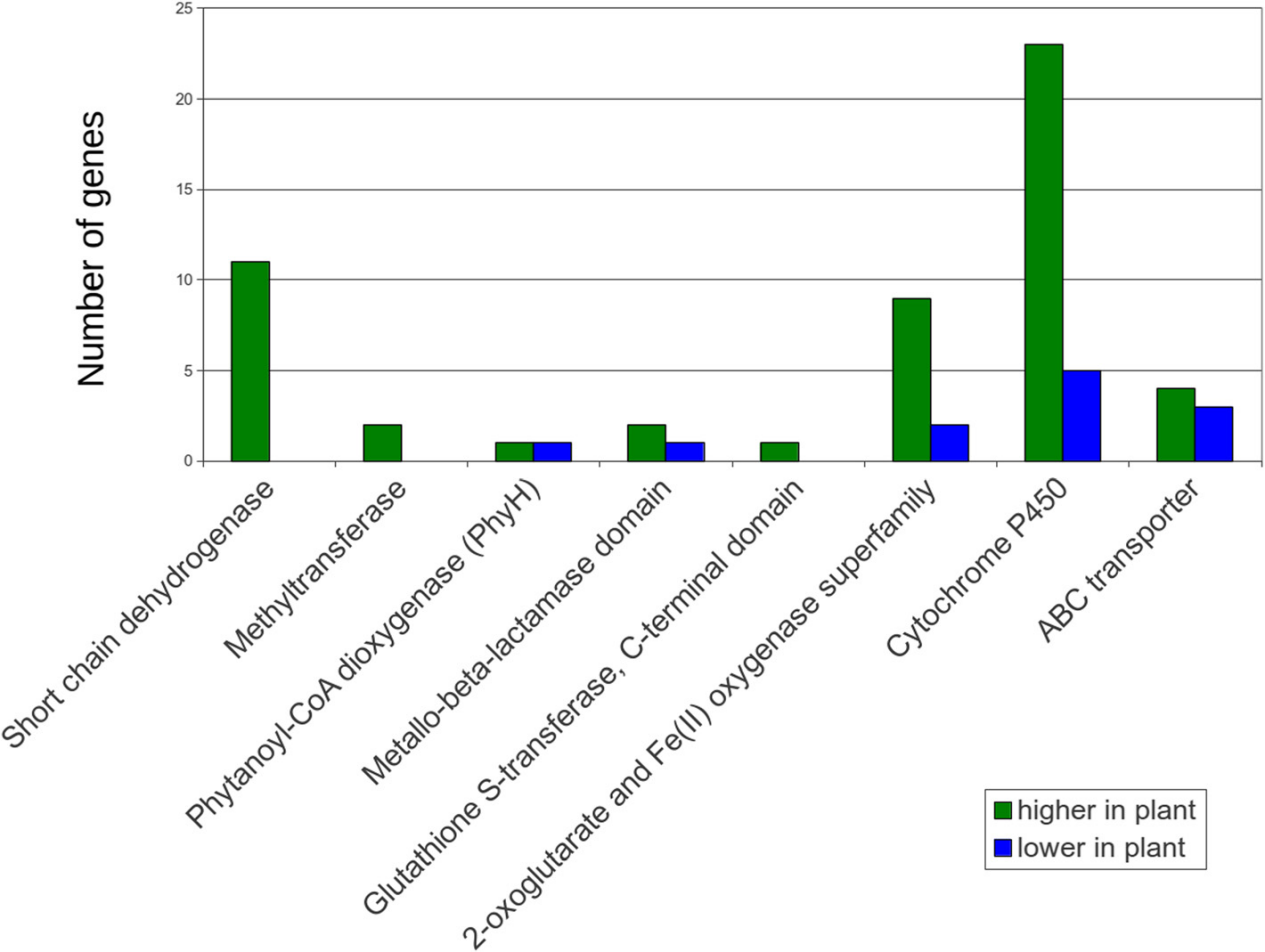
Differentially-regulated genes identified through domain analysis with homology to secondary metabolite genes. For each of the 802 differentially expressed genes identified from RNA-Seq, a blastp search was done, and conserved protein domains were identified. The figure shows genes with homology to genes commonly involved in secondary metabolism [36]. Green bars = genes more highly expressed in infected leaf tissue relative to mycelium grown in medium; Blue bars = genes with lower expression in infected leaf tissue

The functions of differentially expressed genes based on conserved domains were predicted by identifying homologs using blastp on the National Center for Biotechnology Information’s (NCBI) non-redundant protein sequences database and by identifying conserved domains using NCBI’s Conserved Domain Database (Additional file 4: Table S2) [35]. In cases for which multiple differentially expressed genes had the same conserved domains, the number of genes with those conserved domains with higher expression in infected leaf tissue or in culture was determined. This analysis identified genes involved in secondary metabolism, pathogenesis, and nutrient acquisition as well as other functions. For example, genes commonly involved in secondary metabolism [36], such as those encoding cytochrome P450s, short-chain dehydrogenases, methyltransferases, and 2-oxoglutarate and Fe(II)-dependent oxygenases all had many more genes more highly expressed in infected leaf tissue as compared to growth in culture (Fig. 2). Twenty-three cytochrome P450 genes were more highly expressed in infected plant tissue, and only five had lower expression. Similarly, 11 short-chain dehydrogenase genes were more highly expressed in infected leaf tissue, and none had lower expression (Fig. 2). These findings are consistent with the identification of secondary metabolite genes as among those most highly expressed in the infected plant tissue (Table 1).

**Fig. 3:**
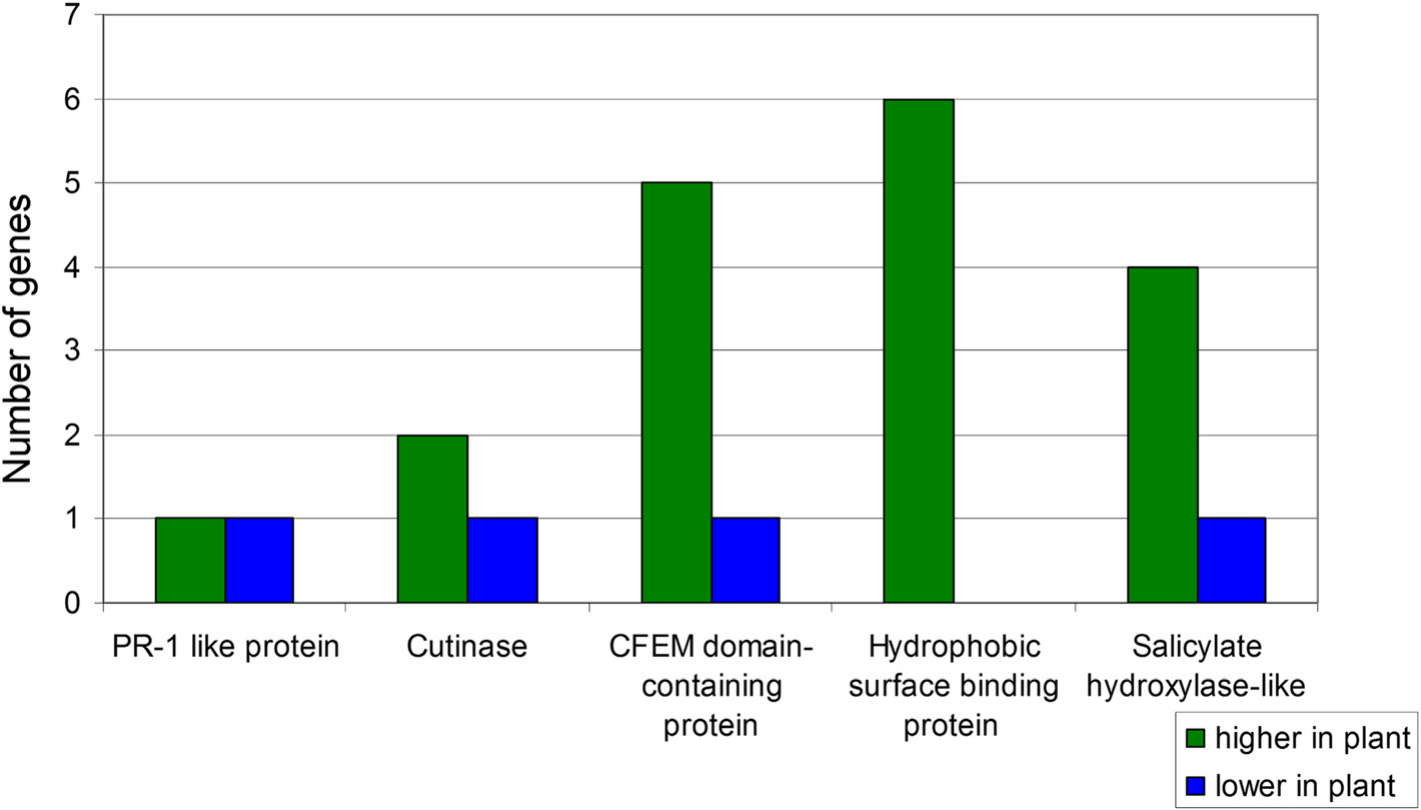
Differentially-regulated genes identified through domain analysis with homology to genes with roles in pathogenesis. For each of the 802 differentially expressed genes identified from RNA-Seq, a blastp search was done, and conserved protein domains were identified. The number of genes with higher expression in the infected leaf tissue or in culture medium from each category of genes with roles in pathogenesis in other species was determined and is indicated in the bar chart. Green bars = genes more highly expressed in infected leaf tissue relative to mycelium grown in culture medium; Blue bars = genes with lower expression in infected leaf tissue

In addition to secondary metabolite genes, types of genes previously implicated in pathogenesis were also identified through the domain analysis (Fig. 3). For example, although one of the most highly expressed genes in culture compared to infected leaf tissue encodes a CFEM domain-containing protein (Table 2), five genes encoding proteins with CFEM domains were more highly expressed in infected leaf tissue, whereas only one had lower expression than in culture. Six genes encoding proteins with conserved Hydrophobic Surface Binding Protein A (HsbA) domains, found in proteins implicated in the recruitment of cutinases important for pathogenicity [37, 38], were found to be more highly expressed in infected leaf tissue, and none were found with lower expression. Two differentially regulated transcripts were identified that encode proteins with homology to Pathogenesis-Related Protein 1 (PR-1), one with higher expression in the infected leaf and one with higher expression in culture (Fig. 3). In plants, PR-1 proteins are synthesized in response to pathogen attack and play roles in defense [39, 40]. In fungal pathogens, however, some PR-1-like proteins have roles in pathogenesis and are required for full virulence [41, 42]. Finally, four salicylate hydroxylase-like genes were more highly expressed in the infected leaf tissue, and one had lower expression (Fig. 3). Salicylic acid is important for plant defense responses [43], and salicylate hydroxylase interferes with defense by degrading salicylic acid to catechol [44]. To further characterize these sequences, blastp analysis was conducted using the *Epichloë festucae* salicylate hydroxylase sequence (Additional file 5: Table S3) [45]. The homolog with the highest similarity (Accession XP_007932011.1) is among the sequences with higher expression in infected leaf tissue, with a log2FC of 3.9 (Additional file 4: Tables S2 and Additional file 5: Table S3).

**Fig. 4:**
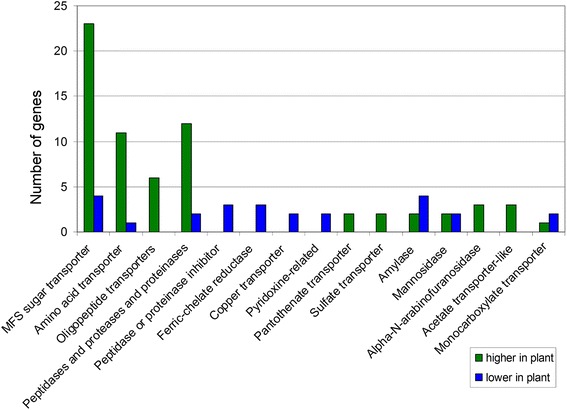
Differentially-regulated genes identified through domain analysis with homology to genes with roles in nutrition. For each of the 802 differentially expressed genes identified from RNA-Seq, a blastp search was done, and conserved protein domains were identified. The number of genes with higher expression in the infected leaf vs. in culture medium from each category of genes with putative roles in response to nutrient levels in the environment was determined and is indicated in the bar chart [112–123]. Green bars = genes with higher expression in infected leaf tissue relative to mycelium grown in medium; Blue bars = genes with lower expression in infected leaf tissue

Domain analysis also showed that differentially expressed genes have possible roles in acquiring nutrients from the environment. For example, 23 MFS sugar transporter genes were more highly expressed and only 4 had lower expression in infected leaf tissue relative to the fungus grown in medium (Fig. 4). More genes encoding peptidases, proteases, proteinases, amino acid transporters, and oligopeptide transporters were more highly expressed in infected leaf tissue, whereas more genes encoding inhibitors of peptidases and proteinases were more highly expressed in culture (Fig. 4). Several ferric-chelate reductase and copper transporter genes were more highly expressed in culture, and none showed higher expression in infected leaf tissue (Fig. 4).

**Fig. 5:**
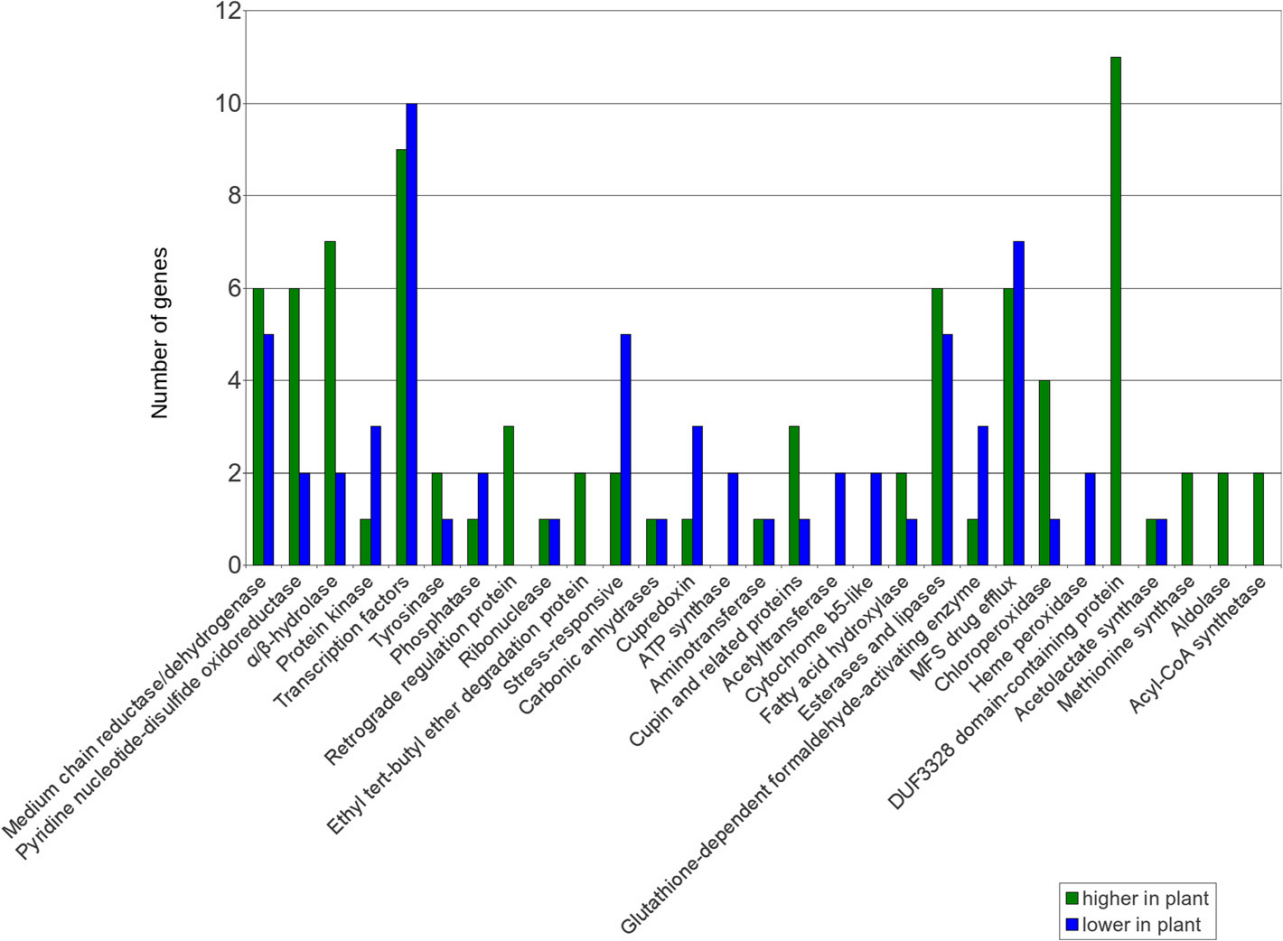
Differentially-regulated genes identified through domain analysis with homology to genes with miscellaneous biological roles. For each of the 802 differentially expressed genes identified from RNA-Seq, a blastp search was done, and conserved protein domains were identified. The number of genes with higher expression in the infected leaf or in culture medium from each category of genes with miscellaneous biological roles was determined and is indicated in the bar chart. Green bars = genes with higher expression in infected leaf tissue relative to mycelium grown in medium; Blue bars = genes with lower expression in infected leaf tissue

Other genes for which a greater number were more highly expressed in the infected leaf tissue were ones that encode proteins with domains for pyridine nucleotide disulfide oxidoreductases, α/β-hydrolases, chloroperoxidases, retrograde regulation proteins, ethyl tert-butyl ether degradation proteins, and proteins with DUF3328 domains (Fig. 5). The difference in expression of transcripts encoding DUF3328-containing proteins was especially dramatic: 11 genes encoding proteins with DUF3328 domains were more highly expressed in infected leaf tissue and none were more highly expressed in culture (Fig. 5). These results agree with the previous finding that two of the 20 genes with highest expression in the infected leaf tissue compared to in culture medium have DUF3328 domains (Table 1). Although the function of DUF3328 domain-containing proteins is unknown, some studies have suggested involvement in sexual reproduction in fungi [46, 47]. In contrast, there were more transcripts encoding protein kinases, cupredoxins, ATP synthases, acetyltransferases, cytochrome b5-like proteins, glutathione-dependent formaldehyde-activating enzymes, heme peroxidases, and genes annotated as stress responsive that were more highly expressed in culture than in the infected leaf (Fig. 5). Many genes encoding transcription factors were differentially expressed, but there were similar numbers of genes with higher expression in the infected leaf tissue (9 genes) versus in medium (10 genes) (Fig. 5). Transcription factors were further characterized based on conserved domains (Additional file 6: Table S4). Overall, the transcription factors encoded by genes more highly expressed in medium had a greater diversity of domains, including the helix-loop-helix, Zn2Cys6, jumonji, bZIP, fungal-specific transcription factor, and NDT80/PhoG-like transcription factor domains (Additional file 6: Table S4). Transcription factors encoded by genes with higher expression in infected leaf tissue had fewer types of domains, and five of these nine transcription factors had only a fungal-specific transcription factor domain (Additional file 6: Table S4).

#### GO annotation of differentially expressed genes

Blast2GO is a tool for associating Gene Ontology (GO) [48] terms with sequences of interest [49, 50]. This program uses blast to find homologs of the input sequences. InterProScan searches against all the European Bioinformatics Institute databases to find protein signatures [51, 52]. GO terms associated with the blast hits are mapped using annotation files from the GO Consortium. Depending on the similarity of the input sequence with the blast hit, as well as the quality of the evidence code, the input sequences are finally annotated with these GO terms [49, 50].

Blast2GO was used with *M. fijiensis* sequences, resulting in 12598 sequences (96.1 % of total sequences) with blast hits (Additional file 7: Figure S3). Gene Ontology (GO) annotations were obtained for 6678 sequences (50.9 % of total sequences) (Additional file 7: Figure S3). After annotations were obtained for each gene, Blast2GO was used for GO enrichment analysis. This analysis identified GO terms that were significantly over-represented from sequences having higher expression in infected leaf tissue or in culture medium, compared to their representation in the total set of genes from the *M. fijiensis* genome. Several GO terms were significantly over-represented in sequences having higher expression in infected leaf tissue (Table 3), whereas no GO terms were found to be over-represented in sequences having higher expression in medium. Oxidoreductase, monooxygenase, dioxygenase, and O-methyltransferase activities were found to be significantly over-represented in sequences having higher expression in infected leaf tissue, which is consistent with our previous results (Fig. 2). Carbohydrate transport was also found to be over-represented, which is consistent with our finding that more sugar transporters had higher than lower expression in infected leaf tissue (Fig. 4). Of the 33 genes annotated by GO as encoding iron ion binding activities, 23 correspond to genes shown by blastp and conserved domain analysis to encode cytochrome P450s, which is consistent with the finding in Fig. 2 that more genes encoding cytochrome P450s are more highly expressed in infected leaf tissue.

**Table 3:** Over-represented GO terms in sequences having higher expression in infected leaf tissue

GO-ID	Term	Category	FDR	*P*-Value
GO:0016491	oxidoreductase activity	Molecular Function	1.31E-16	1.69E-20
GO:0055114	oxidation-reduction process	Molecular Process	1.49E-14	3.85E-18
GO:0004497	monooxygenase activity	Molecular Function	1.11E-13	4.29E-17
GO:0016705	oxidoreductase activity, acting on paired donors, with incorporation or reduction of molecular oxygen	Molecular Function	1.84E-12	9.51E-16
GO:0005506	iron ion binding	Molecular Function	2.61E-12	1.68E-15
GO:0020037	heme binding	Molecular Function	2.35E-08	2.12E-11
GO:0046906	tetrapyrrole binding	Molecular Function	2.35E-08	2.12E-11
GO:0055085	transmembrane transport	Molecular Process	1.38E-04	1.42E-07
GO:0016021	integral component of membrane	Cellular Component	1.83E-04	2.12E-07
GO:0031224	intrinsic component of membrane	Cellular Component	2.71E-04	3.49E-07
GO:0003824	catalytic activity	Molecular Function	3.62E-03	5.12E-06
GO:0046914	transition metal ion binding	Molecular Function	1.29E-02	2.00E-05
GO:0051213	dioxygenase activity	Molecular Function	1.54E-02	2.58E-05
GO:0008643	carbohydrate transport	Molecular Process	4.86E-02	9.18E-05
GO:0008171	O-methyltransferase activity	Molecular Function	4.86E-02	9.38E-05

Table indicates the over-represented GO term, its ID number, its category (Molecular Function, Molecular Process, or Cellular Component), false discovery rate (FDR), and *p*-value

#### CAZy annotation of differentially expressed genes

**Fig. 6:**
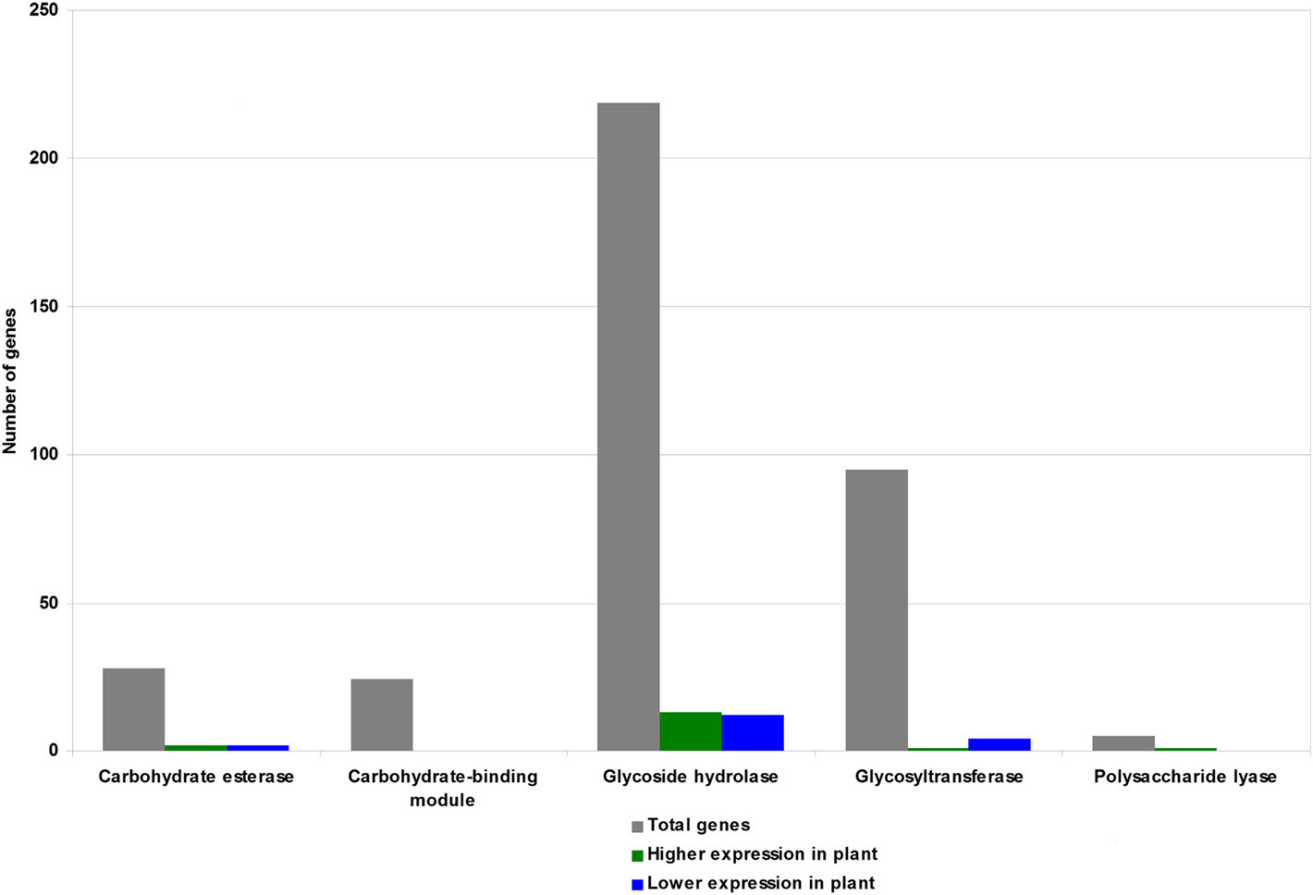
Number of genes with CAZy annotations. Indicated are the number of genes with each CAZy annotation within: Gray = All *M. fijiensis* genes; Green = List of genes having higher expression in infected leaf tissue; Blue = List of genes having higher expression during growth in medium

In addition to the analysis by GO annotation done by Blast2GO, the differentially expressed genes were also analyzed by CAZy annotations. The CAZy database describes families of enzymes that create, degrade, or modify glycosidic bonds [53]. Genes with CAZy annotations were compared with the list of differentially expressed genes. The majority of genes encoding enzymes in the CAZy database were not differentially regulated (Fig. 6). For those that were, the analysis revealed similar numbers of genes with higher or lower expression in plant tissue. These included genes encoding carbohydrate esterases, glycoside hydrolases, and polysaccharide lyases; slightly more glycosyl transferases were more highly expressed in culture than in infected leaf tissue. No sequences annotated as having carbohydrate binding modules were differentially expressed (Fig. 6). Differentially expressed CAZymes were further analyzed (Additional file 8: Table S5). This analysis revealed that differentially expressed CAZymes were distributed to two carbohydrate esterase families, 16 glycoside hydrolase families, and four glycosyl transferase families (Additional file 8: Table S5), but no patterns in families with higher expression in either condition were apparent.

#### Effector protein predictions

Pathogenic fungi secrete effector proteins into the plant apoplast that modulate host physiology and suppress or otherwise protect the pathogen from host defenses [54]. Secreted proteins can be predicted by the presence of a signal peptide, which are N-terminal peptides that target proteins for translocation across the endoplasmic reticulum membrane and that are cleaved off during the translocation process [55]. The overwhelming majority of known fungal effectors are less than 300 amino acids in length after the signal peptide is cleaved [54]. Further, most avirulence effectors are cysteine-rich [56], because the disulfide bonds from the cysteines provide stability against plant proteases in the apoplast [57].

To identify small, cysteine-rich, secreted proteins, the program SignalP 4.1 [58] was first used to predict *M. fijiensis* protein sequences that contain a signal peptide. From this analysis, 863 protein sequences (7 % of total sequences) were predicted to contain a signal peptide. SignalP 4.1 was also used to generate predictions of the mature protein sequences once the signal peptides are cleaved. Of the 863 mature protein sequences, 394 were less than 300 amino acids in length, and 231 of these sequences were considered cysteine-rich, containing four or more cysteine residues.

Genes encoding 40 of the 231 short, cysteine-rich, secreted proteins were differentially expressed in our RNA-Seq analysis (Additional file 9: Table S6). Thirty were more highly expressed in infected leaf tissue, whereas only 10 were more highly expressed in culture (Additional file 9: Table S6). Of the protein sequences encoded by the 30 transcripts that were more highly expressed in infected leaf tissue, six had conserved domains: one had a PR-1-like protein domain, two were predicted to be cutinases, one had a CFEM domain, one had a DUF3328 domain, and one had a globin-like domain (Additional file 9: Table S6). Two identified as hypothetical were among those with the highest expression in infected leaf tissue compared to culture medium (Table 1, Additional file 9: Table S6). For the 10 short, cysteine-rich, secreted proteins whose transcripts were more highly expressed in culture, two had conserved domains: one had a CFEM domain, and the other had a serine/threonine phosphatase domain (Additional file 9: Table S6). The CFEM domain-encoding transcript was also identified in the list of genes with highest expression in culture medium compared to infected leaf tissue (Table 2, Additional file 9: Table S6). For the remaining 32 differentially expressed genes encoding short, cysteine-rich, secreted proteins, no significant homology to characterized protein sequences and no predicted conserved domains could be identified (Additional file 9: Table S6). Overall, 16 of the 30 differentially expressed putative effector genes with higher expression in the infected leaf tissue had homologs restricted to species within the Mycosphaerellaceae (Additional file 9: Table S6); others had homologs outside this family. This result is consistent with the observation that many fungal effectors have a restricted phylogenetic distribution [6].

Homologs of the Ecp2, Ecp6 and Avr4 effectors of the tomato pathogen *Cladosporium fulvum* have been identified in *M. fijiensis*, and the *M. fijiensis* Ecp2 and Avr4 effectors are recognized by tomato R proteins that normally recognize the *C. fulvum* Ecp2 and Avr4 effectors [8, 9]. In *C. fulvum*, Avr4 and Ecp6 bind chitin and protect fungal cell walls from plant chitinases [59]. The function of Ecp2 is not known [9]. In our dataset, none of the genes were strongly differentially expressed: *MfAvr4* had a log2FC of -0.8 with an adjusted *p*-value of 0.05, and the expression of *MfEcp2* and *MfEcp6* was unchanged between the two conditions (Additional file 3: Table S1). This result may be due to our focus on the necrotrophic phase in our transcriptome analysis.

### Identification of differentially expressed gene clusters

To identify potential gene clusters, we searched for loci in the genome with at least three adjacent genes that were similarly differentially expressed. Using this method, 16 gene clusters were identified with higher expression in infected leaf tissue, and 5 clusters were identified with lower expression in infected leaf tissue. Three of the putative clusters with higher expression in infected leaf tissue encode polyketide pathways, and have been previously described [17]. The remaining 18 clusters are detailed in Additional file 10: Table S7.

**Fig. 7:**
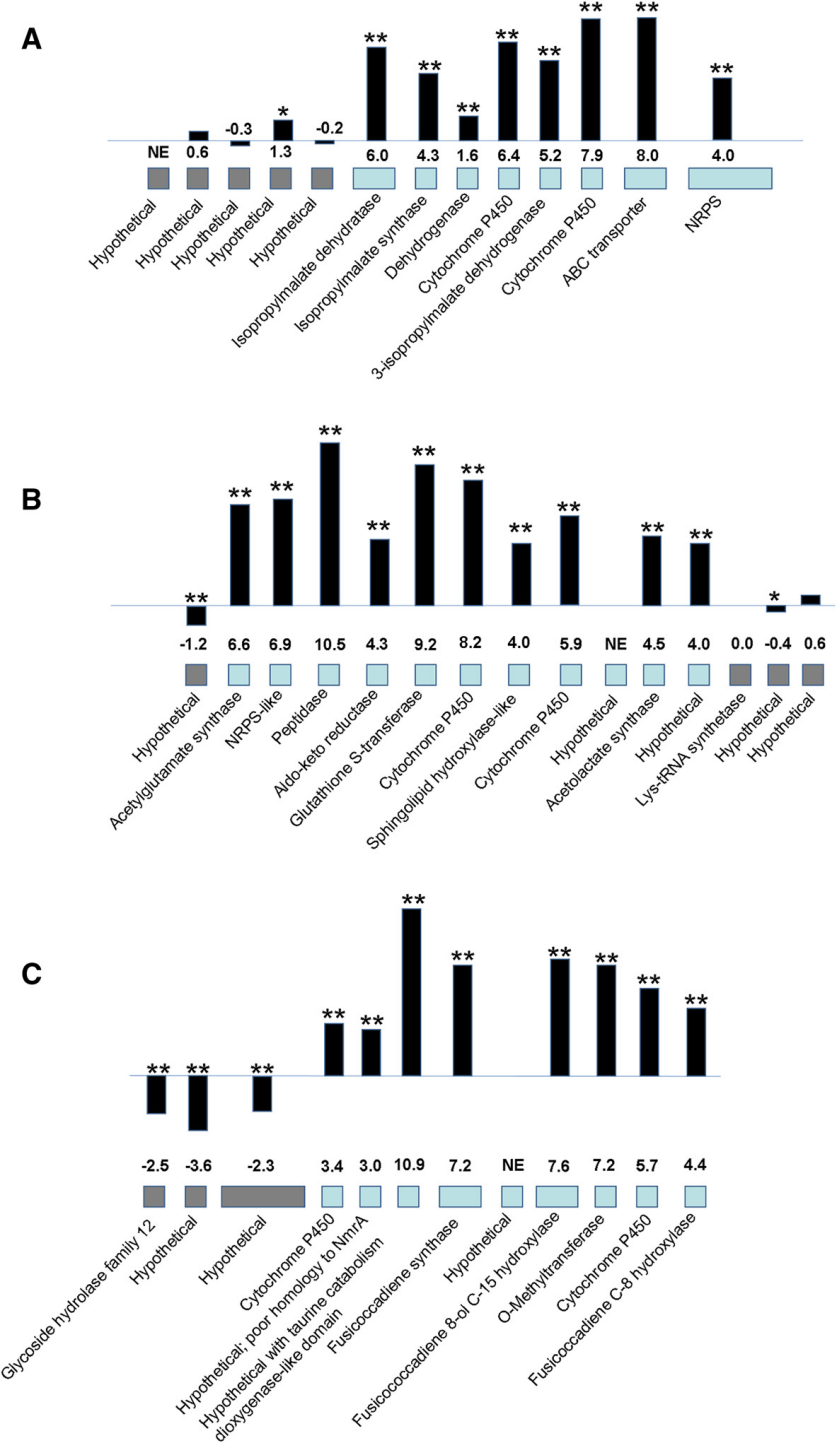
Non-ribosomal peptide synthase (NRPS), NRPS-like, and fusicoccane clusters with higher expression in infected leaf tissue. The NRPS or NRPS-like gene is shown along with its neighboring genes in the *M. fijiensis* genome. The description of each gene as determined by blastp of the corresponding protein is shown along with its log2FC value of expression in infected leaf tissue versus expression in liquid medium. Blue boxes indicate genes in putative cluster; gray boxes indicate genes flanking the cluster. Black bars are proportional to the log2FC value. Gene expression differences that are significant at *p* < 0.01 are shown with two asterisks above the corresponding bar, and those significant at *p* < 0.05 are shown with a single asterisk. NE = no expression detected. **a** NRPS gene cluster on scaffold 7; **b** NRPS-like gene cluster on scaffold 4; **c** Fusicoccane gene cluster on scaffold 2

Genes encoding secondary metabolite pathways are often clustered in fungal genomes [36], and our cluster analysis supports the importance of secondary metabolism in *M. fijiensis* disease development. Two of the genes shown in Table 1 with highest expression in infected leaf tissue compared to culture medium are part of a polyketide synthase cluster (*PKS7-1*) previously described, and two other PKS clusters were also previously shown to be more strongly expressed in infected leaf tissue [17]. Of the remaining 13 clusters identified with higher expression in infected leaf tissue, two have genes similar to non-ribosomal peptide synthases (NRPS). One of these is an NRPS on scaffold 7 (Fig. 7a). Adjacent genes encoding an ATP-Binding Cassette (ABC) transporter, two cytochrome P450s, a 3-isopropylmalate dehydrogenase, a glyoxylate/hydroxypyruvate reductase, an α-isopropylmalate synthase, and a 3-isopropylmalate dehydratase also showed higher expression in infected leaf tissue (Fig. 7a). A blastp search revealed that the closest homologs of this NRPS are in the related banana pathogens *Mycosphaerella musicola* and *Mycosphaerella eumusae*; both of these species have homologs with 60 % sequence similarity (Additional file 10: Table S7A). Aside from the homologs in *M. musicola* and *M. eumusae*, none of the other top 15 homologs were found in Dothideomycete species (Additional file 10: Table S7A). The closest characterized homolog is a destruxin synthetase from *Metarhizium guizhouense*, with 53 % sequence similarity to the *M. fijiensis* NRPS (Additional file 10: Table S7A). Destruxins are cyclic hexadepsipeptides produced by insect pathogens such as *Metarhizium* spp., *Aschersonia* spp., and *Beauveria felina* [60–62]. In these pathosystems, destruxins act insecticidally. Destruxins have also been identified from the plant pathogens *Alternaria brassicae* and *Ophiosphaerella herpotricha*, and are phytotoxic to some plant species [63–66]. In addition to the NRPS gene cluster on scaffold 7, there is a gene cluster containing an NRPS-like gene on scaffold 4 (Fig. 7b). While a true NRPS enzyme must contain a condensation domain, an adenylation domain, and a phosphopantetheine attachment site [67], the NRPS-like enzyme encoded on scaffold 4 was predicted to contain an adenylation domain, but no condensation domain or phosphopantetheine attachment site (Additional file 10: Table S7B). Other genes in this cluster that were similarly more highly expressed in infected leaf tissue include genes encoding an N-acetylglutamate synthase, a peptidase, an aldo/keto reductase, a glutathione S-transferase, two cytochrome P450s, and a sphingolipid hydroxylase-like protein (Fig. 7b). A blastp search of the *M. fijiensis* NRPS-like sequence against the NCBI non-redundant protein sequence database revealed that *M. musicola* has a close homolog with 87 % sequence similarity, but *M. eumusae* does not (Additional file 10: Table S7B). There were no close homologs of this NRPS-like sequence for which a function has been described (Additional file 10: Table S7B).

**Fig. 8:**
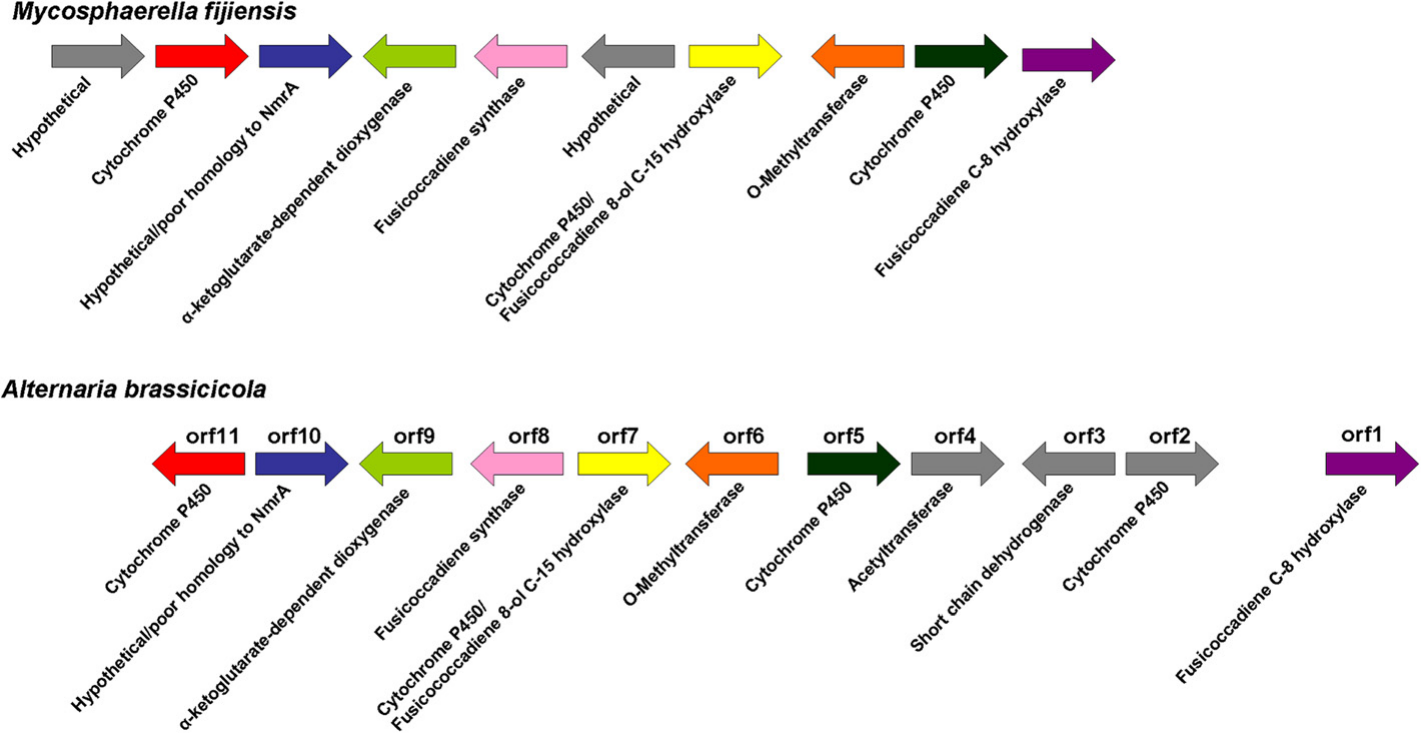
Comparison of *M. fijiensis* and *Alternaria brassicicola* fusicoccane biosynthetic clusters. Genes in the *M. fijiensis* fusicoccane biosynthetic cluster are shown compared to those in the *A. brassicicola* cluster. Orientation of each gene is indicated by the direction of each arrow. Genes encoding proteins with similar functions are indicated by the same color, and genes not shared between the two clusters are shown in gray

Another gene cluster with higher expression in the infected leaf tissue is one with genes having homology to fusicoccane biosynthetic genes (Fig. 7c). Fusicoccanes are diterpenoids with a variety of effects on plant physiology, including opening of stomata [68]. A blastp search was performed of the *M. fijiensis* fusicoccadiene synthase using the non-redundant protein sequence database through NCBI, which identified the best homolog as being from *Alternaria brassicicola* with 83 % sequence similarity (Additional file 10: Tables S7C and Additional file 11: Table S8); this enzyme catalyzes the first step in the synthesis of a fusicoccane called brassicicene C [69]. The top homologs (Additional file 11: Table S8) were used to create a phylogenetic tree, which showed a bootstrap value of 100 for the relationship between the *M. fijiensis* fusicoccadiene synthase and the *A. brassicicola* and *Bipolaris victoriae* fusicoccadiene synthases (Additional file 12: Figure S4). None of the top homologs identified were from Mycosphaerellaceae species (Additional file 11: Table S8), even though genome sequences from several members of this family are publicly available on NCBI, including very close relatives of *M. fijiensis* such as *M. musicola* and *M. eumusae* (NCBI Genome IDs 43744 and 43743, respectively). The gene content and gene orientations for the *M. fijiensis* and *A. brassicicola* fusicoccane biosynthetic clusters were further compared, showing that both clusters contain genes encoding a fusicoccadiene synthase, an α-ketoglutarate-dependent dioxygenase, a hypothetical protein with similarity to nmrA, four cytochrome P450s, and an O-methyltransferase (Fig. 8). The *M. fijiensis* cluster contains a gene encoding a hypothetical protein which the *A. brassicicola* cluster does not have, and the *A. brassicicola* cluster contains genes that the *M. fijiensis* cluster lacks, including: genes encoding a cytochrome P450, a short-chain dehydrogenase, and an acetyltransferase. Gene orientation is largely conserved, with only a cytochrome P450 on one end of the cluster being in different orientations between the two clusters. These results suggest that *M. fijiensis* may produce a fusicoccane very similar, though not identical, to brassicicene C.

**Fig. 9:**
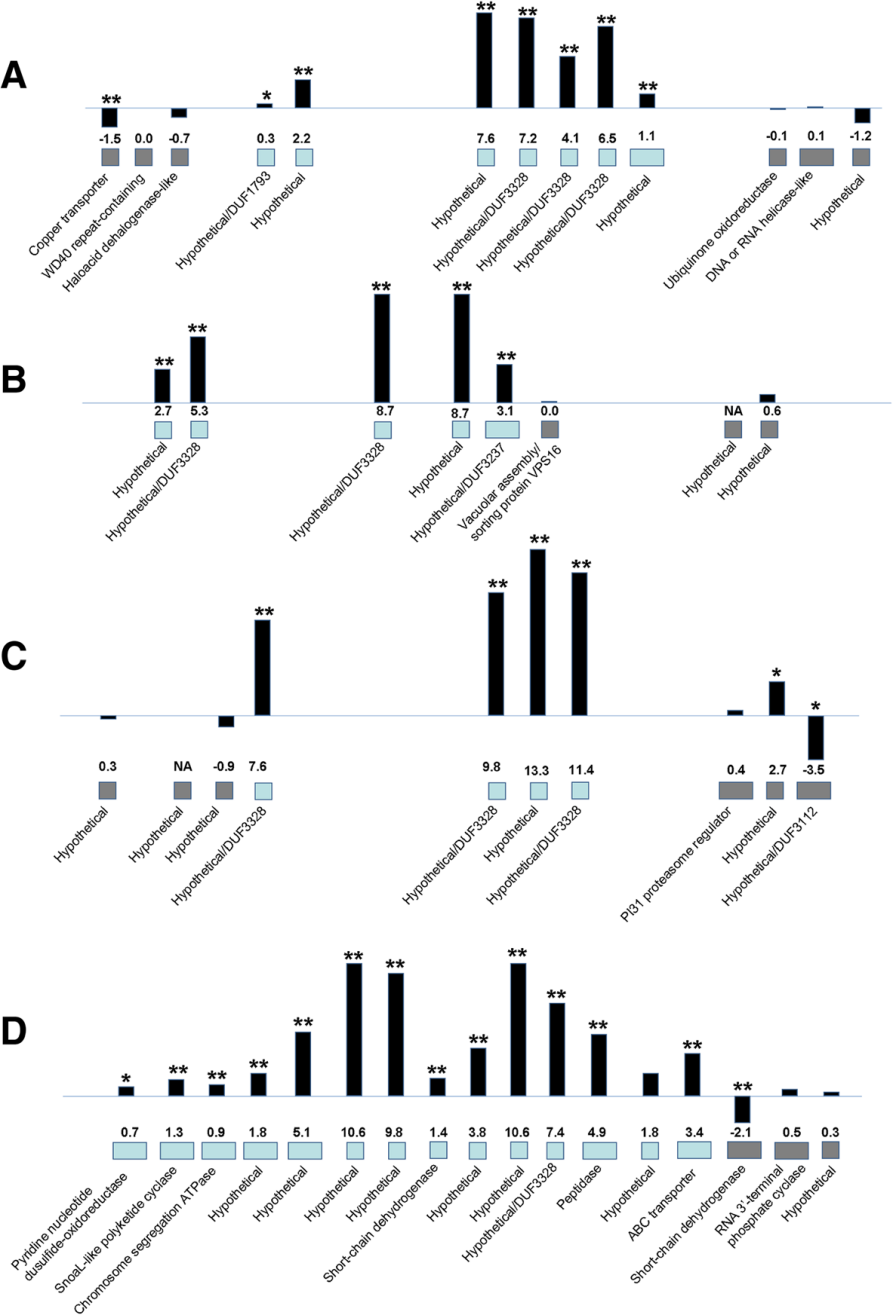
Gene clusters encoding Domain of Unknown Function (DUF) 3328-containing proteins. Gene clusters with higher expression in infected tissue are shown, which contain genes encoding DUF3328 proteins. The description of each gene as determined by blastp of the corresponding protein is shown along with its log2FC value of expression in infected leaf tissue versus expression in liquid medium. Blue boxes indicate genes in putative cluster; gray boxes indicate genes flanking the cluster. Black bars are proportional to the log2FC value. Gene expression differences that are significant at *p* < 0.01 are shown with two asterisks above the corresponding bar, and those significant at *p* < 0.05 are shown with a single asterisk. NE = no expression detected. **a** Gene cluster on scaffold 12; **b** Gene cluster on scaffold 2; **c** Gene cluster on scaffold 9; **d** Gene cluster on scaffold 3

Interestingly, four gene clusters with higher expression in infected leaf tissue include genes encoding DUF3328-containing proteins (Fig. 9, Additional file 10: Table S7D-G). These gene clusters include from one to three DUF3328 genes, and considered together, these clusters contain 9 of the 11 DUF3328 genes with higher expression in infected leaf tissue (Figs. 5 and 9, Additional file 4: Table S2). As noted earlier, the function of DUF3328 domain-containing proteins is unknown, although some studies have suggested involvement in fungal sexual reproduction [46, 47].

Two gene clusters with higher expression in infected leaf tissue, on scaffolds 7 and 21, consist almost entirely of genes encoding hypothetical proteins (Additional file 10: Table S7H-I and Additional file 13: Figure S5A-B). The other clusters contain genes with putative functions, but their roles as gene clusters are unclear (Additional file 10: Table S7J-M and Additional file 13: Figure S5C-F). For example, one cluster contains several genes commonly found in secondary metabolite gene clusters [36] including one encoding a 2-oxoglutarate and Fe(II)-dependent oxygenase superfamily enzyme, two methyltransferases, an oxidoreductase, and a transporter (Additional file 10: Table S7J and Additional file 13: Figure S5C). Another cluster contains genes encoding two hydrophobic surface binding proteins with homology to HsbA and a pyridine nucleotide-disulfide oxidoreductase (Additional file 10: Table S7K and Additional file 13: Figure S5D). HsbA domains are found in proteins implicated in recruitment of cutinases [37, 38], but the role of the oxidoreductase in this gene cluster is unclear.

The five gene clusters with lower expression in infected leaf tissue contain genes encoding proteins with putative functions, but the role of the genes together as a cluster is unclear (Additional file 10: Table S7N-R and Additional file 14: Figure S6). For example, one cluster contains genes encoding a putative cerato-platanin, two hypothetical proteins, and a C2 domain-containing protein (Additional file 10: Table S7N and Additional file 14: Figure S6B). Cerato-platanin was first described as a small, secreted fungal toxin from *Ceratocystis fimbriata* [70]. However, cerato-platanins are produced by both pathogenic and non-pathogenic fungi [71, 72], and they are believed to play multiple roles in fungal biology including promotion of cell wall expansion and hyphal elongation [73, 74]. Though the putative cerato-platanin encoded by this gene cluster may play a role in hyphal elongation in the culture medium, it is unclear whether cerato-platanin would interact with any of the other gene products in this cluster. Another example of a gene cluster with lower expression in the infected leaf tissue contains genes encoding an oxidoreductase, a short-chain dehydrogenase, a choline dehydrogenase, an MFS transporter, an aminoacyl-tRNA ligase, a protein containing ankyrin repeats, two hypothetical proteins, and a sequence with homology to pyoverdine/dityrosine biosynthesis protein (Additional file 10: Table S7O and Additional file 14: Figure S6C). Dityrosine is a cross-linking agent that is present in the cell walls of some fungi and protects against adverse environmental conditions [75, 76]. Dityrosine is present in the outermost wall layer of ascospores in many members of the family Saccharomycetaceae [77]. In *Candida albicans*, dityrosine is also produced along the surface of yeast bud scars and germinated cells [78]. Pyoverdine is a siderophore produced by *Pseudomonas* spp. which consists of a fluorescent chromophore, a peptide chain, and an acyl side chain [79]. While putative functions for many genes in this cluster can be predicted, it is unclear how proteins encoded by this cluster may work together to produce a product.

### Distribution of differentially expressed genes on *M. fijiensis* genome scaffolds

Many fungi have one or more dispensable chromosomes that assist pathogenicity on a particular host, or have other functions that are useful but not strictly necessary for survival [24]. The related species *Mycosphaerella graminicola* has eight dispensable chromosomes which are readily lost when the fungus undergoes meiosis [80]. These dispensable chromosomes from *M. graminicola* have characteristics that distinguish them from the core chromosomes: they are the smallest chromosomes, have the lowest G + C content, have the lowest gene density, have the lowest proportion of genes encoding proteins with PFAM domains, have the highest proportion of repetitive DNA, and have different codon usage [26, 81]. There are 14 scaffolds from the *M. fijiensis* genome that were predicted to also be dispensable, since they have the same characteristics as the dispensable chromosomes from *M. graminicola*: small scaffolds, low G + C content, low gene density, low proportion of genes encoding proteins with PFAM domains, a high proportion of repetitive DNA, and different codon usage [25, 26]. If these scaffolds do correspond to dispensable chromosomes, some may play important roles in pathogenicity as is the case for some other pathogenic fungi [24, 82–86].

**Fig. 10:**
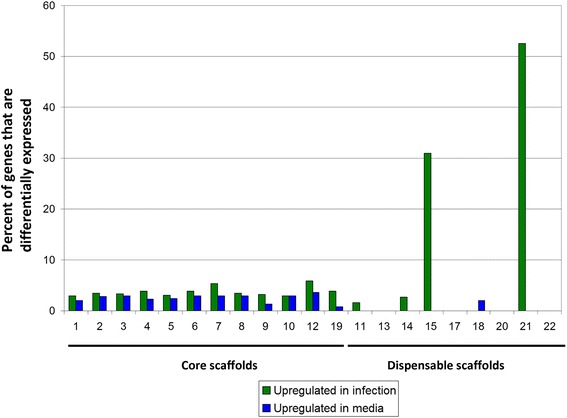
Percent of genes on each *M. fijiensis* scaffold that are differentially expressed. For each *M. fijiensis* genome scaffold with at least 25 predicted genes, the number of genes with higher expression in the infected leaf or in culture is expressed as a percentage of the total number of genes on that scaffold. Predictions of whether the scaffold may represent a core or dispensable scaffold [25, 26] are indicated underneath the scaffold number. Scaffolds were predicted to be dispensable based on the following characteristics: small scaffolds, low G + C content, low gene density, low proportion of genes encoding proteins with PFAM domains, high proportion of repetitive DNA, and different codon usage [25, 26]. Green = Genes more highly expressed in infected leaf tissue; Blue = Genes with lower expression in infected leaf tissue

Scaffolds which contain at least 25 genes and were predicted to be dispensable by Ohm et al [25] are indicated in Fig. 10. To determine whether these putative dispensable scaffolds from *M. fijiensis* have a different percentage of differentially expressed genes compared to the core scaffolds, the differentially expressed genes were sorted based on their scaffold of origin (Fig. 10). This analysis revealed that 31 % (13 genes out of 42) and 52 % (21 genes out of 40) of the genes on the predicted dispensable scaffolds 15 and 21, respectively, were more highly expressed in infected leaf tissue, and no genes on these scaffolds had lower expression (Fig. 10, Additional file 15: Table S9). By contrast, less than 10 % of the genes on the core scaffolds were more highly expressed in infected leaf tissue (Fig. 10). These results suggest that scaffolds 15 and 21 may play roles in pathogenicity. About two-thirds of the predicted proteins encoded by genes on scaffolds 15 and 21 had no blast hits and no conserved domains (61 and 69 %, respectively) (Additional file 15: Table S9 and Additional file 16: Figure S7). For those from scaffolds 15 and 21, respectively, that did have homologs, 80 % and 100 % were in the related banana pathogens *M. musicola* and *M. eumusae* (Additional file 15: Table S9 and Additional file 16: Figure S7)*.* Two gene models from scaffold 15 and one from scaffold 21 had conserved domains: one had a fungal Zn(2)-Cys(6) binuclear cluster domain common in transcription factors, one had a chromosome segregation protein domain, and one had a serine/threonine protein kinase domain. All three of these genes were more highly expressed in infected leaf tissue (Additional file 15: Table S9).

While the other putative dispensable scaffolds did not have as large a proportion of differentially expressed genes as were found on scaffolds 15 and 21, they did have very different patterns of differential expression compared to the core scaffolds (Fig. 10). Each of the core scaffolds had some genes that were more highly expressed in the infected leaf tissue and other genes that were more highly expressed in culture medium (Fig. 10). By contrast, the scaffolds predicted to be dispensable had only genes that were more highly expressed in infected leaf tissue (scaffolds 11, 14, 15, and 21), more highly expressed in culture (scaffold 18), or no differentially expressed genes (scaffolds 13, 17, 20, and 22) (Additional file 3: Tables S1 and Additional file 4: Table S2).

**Fig. 11:**
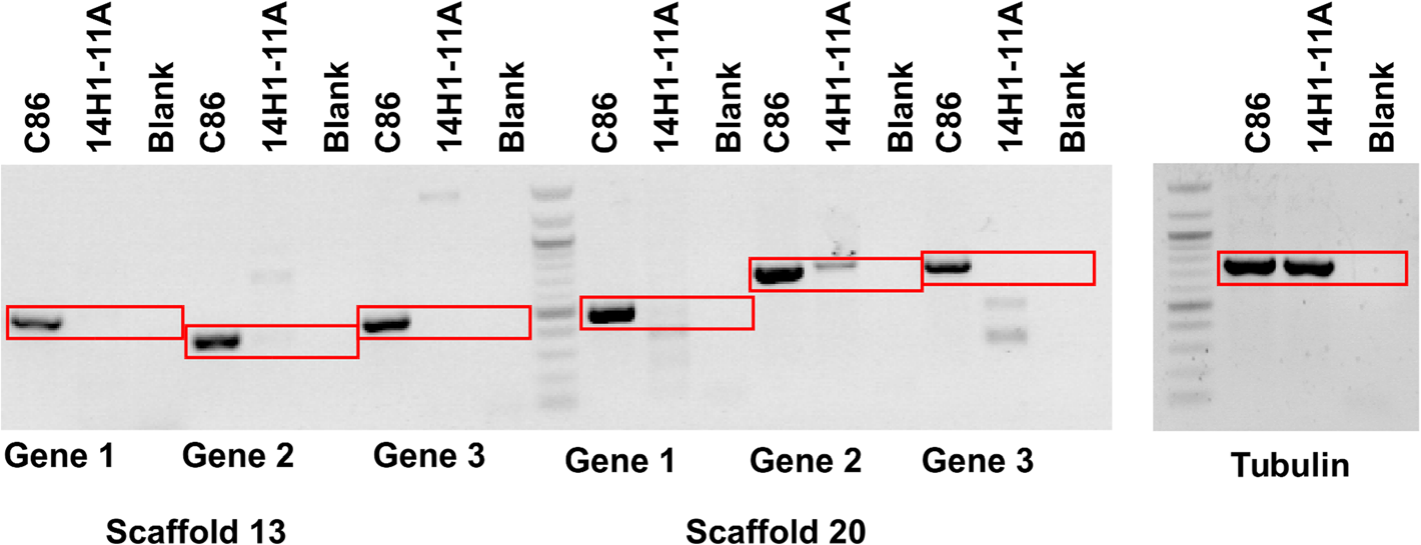
PCR amplification of genes from scaffolds 13 and 20 in isolates 14H1-11A and CIRAD86. PCR amplification was done for three genes encoding hypothetical proteins on scaffolds 13 and 20, as well as β-tubulin as a positive control. Genomic DNA from isolates 14H1-11A (used in this RNA-Seq analysis) and CIRAD86 (genome sequence publicly available) was used as a template for PCR assays, with water used as a negative control. Quick-Load 100 bp DNA ladder (NEB) was used as a molecular weight marker. Red rectangles mark the expected product size for each assay

Although none were differentially expressed between the two conditions, transcripts were detected for 82 % of the 65 genes and 43 % of the 30 genes on scaffolds 17 and 22 (Additional file 3: Tables S1 and Additional file 4: Table S2). In contrast, no transcripts were detected from genes on scaffolds 13 and 20, out of 51 and 29 total genes on these scaffolds, respectively (Additional file 3: Tables S1 and Additional file 4: Table S2). Since no transcripts were detected from scaffolds 13 and 20, we used PCR to assay isolate 14H1-11A (used in the RNA-Seq analysis) for genes on these scaffolds in order to determine whether these scaffolds are present in this isolate. The PCR assays were done for three hypothetical genes on each of scaffolds 13 and 20, and β-tubulin as a positive control. Genomic DNA extracted from isolates 14H1-11A (the isolate used for RNA-Seq) and CIRAD86 (the isolate for which the reference genome is available) was used as a template. This analysis revealed that while the β-tubulin PCR assay resulted in equally strong bands for both isolates, PCR amplifications of all genes on scaffolds 13 and 20 resulted in strong bands for isolate CIRAD86 only, whereas only one gene on scaffold 20 yielded a comparably sized, though fainter, band for isolate 14H1-11A (Fig. 11).

From the lack of transcripts detected from scaffold 13 as well as the lack of amplification of scaffold 13 genes in our PCR amplification of genomic DNA from isolate 14H1-11A, we have no evidence that scaffold 13 is present in isolate 14H1-11A. Results are less clear for scaffold 20. As noted, no transcripts were identified for this scaffold in our transcriptome analysis, and two out of the three genes tested by PCR were not amplified. However, a faint band was amplified from 14H1-11A for gene 2 on this scaffold (Fig. 11). This band was gel purified and sequenced for both isolates, and the sequences were used for a blastn search against the *M. fijiensis* genome. The blast search showed that the DNA sequence for isolate 14H1-11A has 83 % identity with the published sequence from isolate CIRAD86 (Table 4). A blastx search was then done of the isolate 14H1-11A translated nucleotide sequence to align this predicted protein sequence against the published sequence from isolate CIRAD86. This analysis revealed that the changes in nucleotide sequence result in several internal stop codons in isolate 14H1-11A (Additional file 17: Figure S8), and therefore 14H1-11A is unlikely to produce a functional protein product. Scaffold 20 may be present but diverged in isolate 14H1-11A, or it may be that only part of that scaffold is present.

**Table 4:** Blast hits of scaffold 20 gene amplified from both isolates CIRAD86 and 14H1-11A (gene 2 in Fig. 11)

Isolate of query sequence	Description of hit	Hit Accession	Bit score	E-value	Percent identity	Gaps
CIRAD86	*Pseudocercospora fijiensis* CIRAD86 hypothetical protein partial mRNA	XM_007934411.1	1129	0	611/611 (100 %)	0/629 (0 %)
14H1-11A	*Pseudocercospora fijiensis* CIRAD86 hypothetical protein partial mRNA	XM_007934411.1	538	5.00E-149	505/609 (83 %)	12/609 (1 %)

Bands of the expected product size were gel purified for isolate CIRAD86 and 14H1-11A, and were sequenced. Blastn searches of both the resulting sequences were done against the *M. fijiensis* genome. The table indicates the isolate from which the sequence was obtained, the description of the hit, the accession of the hit, the bit score, E-value, percent identity, and percent of gaps

## Discussion

Although several studies have investigated banana genes involved in defense against *M. fijiensis* and other *Mycosphaerella* pathogens [29, 30, 32, 33], our work is the first to identify candidate pathogenicity genes based on analysis of the *M. fijiensis* transcriptome during its association with banana. Our analysis identified 802 genes that were differentially expressed in infected leaf tissue compared to culture medium. Of these, 483 genes had higher expression in infected leaf tissue, and 319 genes had higher expression in medium.

Secondary metabolic pathways have long been suspected as important during the interaction between this fungus and banana [1, 11, 12, 87–90], and we found that two of the genes with the highest expression in infected leaf tissue compared to culture medium (Table 1) were from the previously described *PKS7-1* polyketide gene cluster [17], whose product and function are currently unknown. Many types of genes commonly involved in secondary metabolism were also found to have higher expression in infected leaf tissue compared to growth in medium. These genes include ones encoding cytochrome P450s, short-chain dehydrogenases, and oxidoreductases in the 2-oxoglutarate and Fe(II)-dependent oxygenase superfamily (Figs. 2 and 7) [36]. In addition to the three polyketide synthase gene clusters recently described [17], we showed that an NRPS, an NRPS-like, and a fusicoccane gene cluster had higher expression in infected leaf tissue compared to medium (Fig. 7). The closest characterized homolog to the *M. fijiensis* NRPS protein sequence was a destruxin synthase. Some fungal pathogens produce destruxins which are toxic to their insect or plant hosts and are thought to be involved in virulence [60–66]. Further research is needed to identify the product of the NRPS and its possible role in virulence.

Another gene cluster identified with higher expression in the infected leaf tissue is predicted to encode a fusicoccane. Fusicoccanes are diterpenoids produced by a variety of organisms including fungi, plants, and liverworts. They share a common C20 core, but vary on stereochemistry, degree of saturation, and substitutions, which all affect their physiological activity [68]. Some fusicoccanes cause stomatal opening and wilting, affect seed germination and cell elongation, have antibacterial and antifungal activities, and cause inhibition of biological nitrification and of lysophospholipase [91]. The predicted fusicoccane gene cluster in *M. fijiensis* is very similar to a cluster producing brassicicene C from *Alternaria brassicicola*, though it lacks a cytochrome P450, a short-chain dehydrogenase, and an acetyltransferase that the *A. brassicicola* cluster contains (Fig. 8). These differences suggest that the fusicoccane side groups may slightly differ, thus we hypothesize that the cluster encodes a novel fusicoccane*.* No significant antimicrobial activity has been detected from brassicicene, and its phytotoxicity and effect on the pathogenicity of *A. brassicicola* are unknown [92].

Other types of genes previously implicated in pathogenicity in other fungi also had higher expression in the infected leaf tissue. These include genes predicted to encode salicylate hydroxylase-like proteins, hydrophobic surface binding proteins, and CFEM domain-containing proteins (Fig. 3). We showed that the *M. fijiensis* homolog with highest similarity to the *E. festucae* salicylate hydroxylase is among the genes with higher expression in infected leaf tissue (Additional file 5: Table S3). Since salicylic acid is important for plant defense responses [43], and salicylate hydroxylase converts salicylate to catechol [44], production of salicylate hydroxylase by *M. fijiensis* could be a strategy to dampen the salicylic acid defense pathway. In *Aspergillus oryzae*, the hydrophobic surface binding protein HsbA is secreted from fungal tissue and promotes degradation of the hydrophobic compound polybutylene succinate-co-adipate (PBSA) by recruiting cutinase [37]. PBSA is structurally similar to waxes in the plant cuticle [38, 93]; therefore, it is thought that HsbA and related proteins may recruit cutinases important for plant pathogenicity [38]. CFEM domains are cysteine-rich domains that have been identified in proteins important for pathogenicity [34].

We observed that more genes encoding amino acid transporters, oligopeptide transporters, peptidases, proteases and proteinases had higher expression in infected leaf tissue compared to culture medium (Fig. 4). One proteinase gene was among the list of 20 genes with highest expression in infected leaf tissue compared to culture medium (Table 1). Conversely, more peptidase or proteinase inhibitor genes had lower expression in infected leaf tissue (Fig. 4). These results may be expected if the *in planta* environment is lower in nitrogen than the PDB culture medium. In other plant pathogenic fungi and bacteria, low nitrogen conditions are known to induce expression of virulence genes, and it is thought that this reflects nitrogen limitation for the pathogen during its interaction with the plant host [94, 95]. Likewise, more sugar transporters had higher expression in infected leaf tissue compared to medium (Fig. 4). This may reflect a higher sugar concentration in the PDB medium due to added dextrose, and thus a need for higher expression of sugar transporters in the plant environment compared to a relatively sugar-rich medium.

We identified four gene clusters with higher expression in the infected leaf tissue that encode one or more hypothetical proteins with Domain of Unknown Function (DUF) 3328 (Fig. 9). Two DUF3328-encoding genes were among the list of genes with highest expression in the infected leaf tissue compared to medium (Table 1). Although the function of this domain is not known, some studies have associated changes in DUF3328 gene expression with the fungal sexual cycle. In *Sordaria macrospora*, a DUF3328 gene cluster was identified as up-regulated in wild-type protoperithecia compared to protoperithecia of a mutant for sexual reproduction [46]. In *Podospora anserina*, genes encoding DUF3328 proteins were identified as having higher expression in mat- compared to mat + strains [47]. In natural infections where both mating types of *M. fijiensis* are present, pseudothecia develop in infected banana tissue, and ascospores are considered the primary means of pathogen spread [1]. Therefore, although the role of DUF3328 proteins remains unclear, one hypothesis is that they may be important for pseudothecia development in this fungus.

We identified genes predicted to encode small, cysteine-rich, secreted proteins, since these are features common in fungal effectors [54, 56]. We found 30 such genes with higher expression in infected leaf tissue, and 10 with higher expression in medium (Additional file 9: Table S6). Two of these were among the list of genes with highest expression in infected leaf tissue compared to culture medium (Table 1), and one was among the list of genes with highest expression in culture medium compared to infected leaf tissue (Table 2). Among the proteins encoded by the 30 genes with higher expression in infected leaf tissue were proteins implicated in other species as being important for pathogenicity, including cutinases and proteins with PR-1-like and CFEM domains [34, 41, 42, 96]. For 16 of the putative effector genes with higher expression in the infected leaf tissue, all homologs were restricted to species within the Mycosphaerellaceae. This result is consistent with the observation that many fungal effectors have a restricted phylogenetic distribution [6]. Although it has previously been shown that the *M. fijiensis* genome encodes homologs of the *C. fulvum* effectors Avr4, Ecp2 and Ecp6 [8, 9], none of these genes had higher expression in infected leaf tissue compared to culture medium (Additional file 3: Table S1). It is possible that these effectors are important for pathogenicity at different time points, such as the biotrophic phase, than for the necrotrophic phase assayed in this experiment.

Finally, we were able to provide support for the hypothesis that *M. fijiensis* may have dispensable chromosomes, and that some of these may be important for pathogenicity [25, 26]. Ohm et al. showed that the *M. fijiensis* genome has 14 scaffolds with similar characteristics to the dispensable chromosomes from *M. graminicola* [25]. Compared to the rest of the genome, these 14 scaffolds are small, have a lower G + C content, different codon usage, a high proportion of repetitive DNA, a low gene density, and a lower proportion of genes encoding proteins with PFAM domains [25]. We identified two of these putative dispensable scaffolds (15 and 21) for which 31 % and 52 % of the genes, respectively, on the scaffolds had higher expression in infected leaf tissue, and no genes on these scaffolds had higher expression in culture medium. It is still unknown whether these scaffolds correspond to dispensable chromosomes, but our data suggest that they may play a role in pathogenicity. We also identified two more scaffolds (13 and 20) for which no transcripts were detected. PCR assays were unable to amplify the three genes tested on scaffold 13, and two of three genes on scaffold 20 in our isolate 14H1-11A, whereas amplification was possible for all of these genes in isolate CIRAD86, for which the genome sequence is publicly available (NCBI Genome ID 10962) [16] (Fig. 11). Considering our transcriptome and PCR data, we have no evidence that scaffold 13 is present in our isolate, though PCR data show that at least part of scaffold 20 remains. Our data support the hypothesis that scaffold 13 corresponds to a dispensable chromosome that is not required for survival or pathogenicity.

## Conclusions

This study is the first to identify candidate pathogenicity genes of the fungus *Mycosphaerella fijiensis*, based on transcriptome data from this fungus during its necrotrophic phase of infection of banana, compared to during saprophytic growth in culture medium. We showed that gene clusters predicted to synthesize a non-ribosomal peptide and a fusicoccane, as well as many types of genes encoding proteins commonly involved in secondary metabolism, such as cytochrome P450s and short-chain dehydrogenases, have higher expression in infected leaf tissue. We identified several other types of genes with higher expression in infected leaf tissue, including genes predicted to encode salicylate hydroxylase-like proteins, hydrophobic surface binding proteins, CFEM domain-containing proteins, amino acid and sugar transporters, and proteins with DUF3328 domains. Furthermore, we identified two putative dispensable scaffolds with a large proportion of genes with higher expression in infected leaf tissue, suggesting that these scaffolds may play a role in pathogenicity. We also identified two other scaffolds for which no transcripts were detected in either condition, and PCR assays support the hypothesis that at least one of these scaffolds corresponds to a dispensable chromosome that is not required for survival or pathogenicity. Together, these results suggest exciting avenues of further research for an important and understudied pathogen.

## Methods

### Fungal cultures

*M. fijiensis* isolate 14H1-11A, isolated from the FHIA research station in La Lima, Honduras, was kindly provided by Dr. Jean Ristaino (North Carolina State University) and was routinely cultured on Potato Dextrose Agar (PDA) (Difco). Conidial production was induced as described [17, 97].

### Banana tissue culture

‘Grand Nain’ banana tissue culture plants were obtained from Miguel Muñoz (Dole Food Company) and were maintained on modified Murashige and Skoog medium as described [17].

### Inoculation of plants and flasks

Rooted banana plants grown in modified Murashige and Skoog medium were transferred to potting mix under greenhouse conditions. When they were approximately 20 cm in height, plants were transferred to an incubator at 25 °C under cool-white fluorescent light on a 18 h light/6 h dark photoperiod. Plants were inoculated by atomizing with 5.2 × 10^4^/mL conidia in 0.5 % Tween 20 as described [17]. Plants were covered with clear plastic bags to maintain high humidity conditions for 1 week. Symptomatic banana leaf tissue was harvested at 6 weeks post-inoculation by flash-freezing in liquid nitrogen.

For growth in culture, 50 mL flasks of Potato Dextrose Broth (PDB) (Difco) were inoculated with 10 μL of 1.3 × 10^6^/mL conidia and incubated in a rotary shaker at 150 rpm in the dark at 25–30 °C. After one week, mycelium was harvested by filtering through Miracloth, blotting dry, and flash freezing in liquid nitrogen.

### cDNA library construction and Illumina HiSeq sequencing

RNA was isolated using the Spectrum Plant Total RNA kit (Sigma). Samples were DNase treated with DNase I (Roche). Total RNA sequencing was conducted at the Genomic Sciences Laboratory, North Carolina State University. RNA quality was confirmed by gel electrophoresis and an Agilent Bioanalyzer. Strand-specific libraries were created using the NEBNext Ultra Directional library prep kit (New England BioLabs). Single-end 125-base reads were generated using an Illumina HiSeq 2500 platform. Average sequencing yield was 32 million reads per sample.

### Identification of differentially expressed genes

FastQC (http://www.bioinformatics.babraham.ac.uk/projects/fastqc/) was used to verify the quality of RNA-Seq reads. Illumina Truseq adapter sequences and low-quality bases were trimmed using CutAdapt v1.7 with a quality cutoff of 20 and a minimum sequence length of 36 [98].

Sequences were mapped from each sample to both the banana genome, *Musa acuminata* subsp. *malaccensis* double-haploid Pahang [99, 100], and to the *M. fijiensis* genome [16, 26] using Tophatv2.0.9 [101]. Gene expression levels were determined using HTSeqv0.6.0 [102] and the gene annotations available from the Joint Genome Institute (JGI). Differentially expressed genes were identified with an adjusted *p*-value < 0.01, a log2FC value > 3, and a basemean (mean of normalized read counts) > 10, using the program DESeq2 v1.4.5 (Additional file 3: Tables S1 and Additional file 4: Table S2) [103]. Principal component analysis and a volcano plot were done to verify that biological replicates were similar in expression pattern to each other (Additional file 1: Figure S1) and to visualize the distribution of differentially expressed transcripts (Additional file 2: Figure S2).

### Validation by RT-qPCR

Four differentially expressed genes were chosen for validation of RNA-Seq results. Genes were chosen with a basemean value of >150 in both conditions, a log2FC value >3, and an adjusted *p*-value of < 0.01, to select differentially expressed genes that were well-expressed in both conditions.

cDNA was synthesized with iScript Select reverse transcriptase (BioRad). qPCR reactions were performed with iQ SYBR Green SuperMix, in a CFX Connect Real-Time System machine. The program was done with an initial denaturation at 95 °C for 2 min, followed by 50 cycles of 95 °C for 10 s, 57 °C for 30 s, 72 °C for 30 s with a plate read, and then 78 °C for 30 s with a plate read. A melt curve was also done to verify that a single product was formed for each reaction.

Each gene of interest was normalized against two *M. fijiensis*-specific reference genes having the same reaction efficiency as the gene of interest, and fold-change in infected leaf tissue was calculated compared to when grown in liquid medium, using the 2^−ΔΔC^_T_ method [104]. Primer sequences are indicated in Additional file 18: Table S10.

### Prediction of differentially expressed gene functions

#### Blast and conserved domain analysis

For each of the 802 differentially expressed genes, a blastp search was performed using the non-redundant protein sequence database from NCBI (July 2015). NCBI’s Conserved Domain Database [35] was used to predict domains from each protein sequence (Additional file 4: Table S2). To identify the closest *M. fijiensis* homolog of salicylate hydroxylase, a blastp search was done with the *Epichloë festucae* salicylate hydroxylase sequence (Accession = AIY25489.1) [45] against all the *M. fijiensis* isolate CIRAD86 predicted protein sequences in NCBI’s non-redundant protein sequence database (6/1/2016) (Additional file 5: Table S3).

#### CAZy and GO annotations and enrichment analysis

Blast2GO Basic v3.1.3 was used to do blastx v2.2.32+ searches of *M. fijiensis* gene catalog coding sequences from JGI against the NCBI non-redundant protein sequences (nr database), using the default parameters. InterProScan, mapping, and annotation steps were all done with the default parameters. Using the resulting annotation data, Fisher’s Exact Test was used to identify GO terms that were over-represented from sequences having higher expression in infected leaf tissue or in medium, compared to their representation in the total set of genes from the *M. fijiensis* genome.

Genome annotations, including those based on the CAZy database, were downloaded from NCBI for *M. fijiensis* (Genome ID: 10962). Genes with CAZy annotations were compared with the list of differentially expressed genes.

#### Effector protein predictions

*M. fijiensis* Gene Catalog protein models were downloaded from the published genome sequence from JGI, in FASTA format. Signal peptides were predicted from these sequences using SignalP 4.1 [58], with the organism group set to Eukaryotes, and the D-cutoff value set to the default. SignalP 4.1 was also used to make predictions of the mature protein sequences once signal peptides are cleaved. Mature protein sequences were then sorted by length to identify sequences less than 300 amino acids in length. These short, secreted mature peptides were then sorted by cysteine content to identify sequences containing at least four cysteine residues. Differentially expressed genes predicted to be short, secreted, and cysteine rich were noted in Additional file 9: Table S6. Since *M. fijiensis* homologs of the *C. fulvum* effectors Avr4, Ecp2, and Ecp6 have previously been described [8, 9], the expression of the corresponding genes was also reported. JGI gene IDs for *MfAvr4*, *MfEcp2* and *Ecp6* are Mycfi1.estExt_fgenesh1_pg.C_60009, gw1.3.823.1, and estExt_fgenesh1_kg.C_70127, respectively.

#### Phylogenetic tree of fusicoccadiene synthases

Once homologs were identified of the *M. fijiensis* fusicoccadiene synthase using blastp, the *M. fijiensis* sequence and its top 49 homologs (Additional file 11: Table S8) were aligned using MUSCLE v3.8.31 [105] in Mesquite v3.04 [106]. ModelGenerator v0.85 [107] was used to select the best substitution model using the Aikaike and and Bayesian Information Criteria. RaxmlGUI v1.3.1 [108] was used to create a maximum likelihood tree using the JTT + I + G substitution model [109] with slow bootstrap, the autoMRE function, and no outgroup.

### Identification of gene clusters

To identify clusters of differentially expressed genes and to analyze how differentially expressed genes are distributed across the *M. fijiensis* genome scaffolds, scaffold number and position on the scaffold was downloaded from JGI, and matched to each gene ID. To identify clusters of differentially expressed genes, loci were identified that contain at least three adjacent, similarly differentially expressed genes (Figs. 7 and 9, Additional file 10: Table S7, Additional file 13: Figures S5 and Additional file 14: Figure S6).

### Distribution of differentially expressed genes across genome scaffolds

To analyze the distribution of differentially expressed genes across scaffolds, differentially expressed genes were sorted according to scaffold number, and the number of differentially expressed genes on a scaffold was compared to the total number of genes on the scaffold (Fig. 10). For proteins encoded by genes on scaffolds 15 and 21, blastp searches were done using NCBI’s non-redundant protein database (4/26/2016) to identify conserved domains and the top 10 homologs by bitscore of each sequence of interest (maximum E-value = 1 × 10^−5^) (Additional file 15: Table S9 and Additional file 16: Figure S7).

To determine whether scaffolds 13 and 20 are present in isolate 14H1-11A, three genes were chosen on scaffold 13 and scaffold 20 for PCR assays. Genomic DNA was extracted from isolate 14H1-11A and CIRAD86 using DNeasy Plant Mini Kit (Qiagen), according to manufacturer’s instructions. PCR amplifications were done using OneTaq 2X Master Mix with Standard Buffer (NEB), according to manufacturer’s instructions with 35 cycles, with primer sequences, annealing temperatures, extension times, and expected product sizes indicated in Additional file 19: Table S11. To obtain the sequence of the gene on scaffold 20 which amplified for both isolates (accession XM_007934411.1), the high fidelity DNA polymerase iProof (Bio-Rad) was used to amplify this gene from both isolates, according to manufacturer’s instructions with an annealing temperature of 60 °C, an extension time of 20 s, and 35 cycles. The band was gel purified from both isolates using a QIAquick gel extraction kit (Qiagen), and Sanger sequencing was done by Eton Bioscience using the forward primer (Additional file 19: Table S11). A blastn search was done using NCBI’s nucleotide collection database for each sequence against the *M. fijiensis* genome to verify amplification of the correct gene (Table 4). A blastx search was also done with the sequence from isolate 14H1-11A to determine how changes in nucleotide sequence in this isolate affect the sequence of the predicted protein. The program Chimera v1.10.2 [110] was used to display the sequence alignment and the degree of conservation for each residue in the alignment.

## Additional files

Additional file 1:
**Figure S1.** Two-dimensional principal component analysis of infected leaf samples versus mycelium grown in liquid culture. Infected leaf samples are shown as green dots, and samples of mycelium grown in PDB medium are shown as blue dots. (TIF 67 kb) Additional file 2:
**Figure S2.** Volcano plot showing the distribution of differentially expressed sequences. The horizontal axis shows the log_2_ fold change for expression in infected leaf tissue versus the fungus grown in liquid medium, and the vertical axis shows the -log_10_ (*p*-value). Each transcript is represented by a dot, which is colored pink for each of the differentially expressed transcripts listed in Additional file 4: Table S2, and colored blue for transcripts that are not differentially expressed. (TIF 154 kb) Additional file 3:
**Table S1.** DESeq2 statistics for each gene in the *M. fijiensis* genome. For each gene, the table indicates the JGI protein and gene IDs, the location of each gene on the scaffold, the baseMean value (mean of normalized read counts), the log_2_ fold change (log2FC), the standard error of the log_2_ fold change estimate (lfcSE), the Wald statistic (stat), the *p*-value, and the *p*-value adjusted for multiple testing (adjusted *p*-value). (XLS 3065 kb) Additional file 4:
**Table S2.** Differentially expressed genes in infected leaf tissue versus mycelium grown in liquid medium. For each gene, the table includes the JGI protein and gene IDs, the location of each gene on the scaffold, the baseMean value (mean of normalized read counts), the log_2_ fold change (log2FC), the standard error of the log_2_ fold change estimate (lfcSE), the Wald statistic (stat), the *p*-value, the *p*-value adjusted for multiple testing (adjusted *p*-value), a summary of blastp results using the NCBI nr protein sequence database, and the conserved domains identified using NCBI’s Conserved Domain Database. (XLS 618 kb) Additional file 5:
**Table S3.** Identification of *M. fijiensis* salicylate hydroxylase homolog. The *Epichloë festucae* salicylate hydroxylase sequence (accession number = AIY25489.1) [45] was used to do a blastp search against the *M. fijiensis* protein sequences using the NCBI non-redundant protein sequence database. For each of the top 10 hits, the table includes a link to the NCBI accession, JGI gene and protein IDs, bitscore, E-value, and percent identity and similarity. Protein sequences corresponding to genes with higher expression in infected leaf tissue are colored green. (XLS 37 kb) Additional file 6:
**Table S4.** Differentially expressed transcription factors in infected leaf tissue versus mycelium grown in liquid medium. For each differentially expressed transcription factor gene, the table indicates the conserved domains identified using NCBI’s Conserved Domain Database, a summary of blastp results using the NCBI nr protein sequence database, and whether the transcription factor is present in one of the gene clusters that were identified in Additional file 10: Table S7 and [17]. The Table also indicates the JGI gene and protein IDs, the location of the gene on the scaffold, the baseMean value (mean of normalized read counts), the log_2_ fold change (log2FC), the standard error of the log_2_ fold change estimate (lfcSE), the Wald statistic (stat), the *p*-value, and the *p*-value adjusted for multiple testing (adjusted *p*-value). Genes with higher expression in medium are colored in blue, and genes with higher expression in infected leaf tissue are colored in green. (XLS 41 kb) Additional file 7:
**Figure S3.** Annotation results of all predicted genes in the *M. fijiensis* genome, using Blast2GO. Red = Sequences for which blast analysis was done, but no hits were found. Orange = Sequences for which blast analysis was done and hits were found, but no GO terms were mapped to the hits. Green = Sequences for which blast analysis was done and GO terms were successfully mapped to the hits. Blue = Query sequences to which GO terms were successfully annotated. Pink = Sequences for which only the InterProScan results were obtained. (TIF 256 kb) Additional file 8:
**Table S5.** Analysis of CAZyme families. Table shows (A) a summary of all the CAZyme families with differentially expressed genes, and (B) annotations for each gene. A) Summary of all the CAZyme families with differentially expressed genes, and numbers of CAZymes in each family with higher expression in the medium versus in infected leaf tissue. B) For each of the differentially expressed CAZymes, the table indicates the CAZy annotation, the conserved domains identified using NCBI’s Conserved Domain Database, a summary of blastp results using the NCBI nr protein sequence database, JGI gene and protein IDs, the NCBI accession, the location of the gene on the scaffold, the baseMean value (mean of normalized read counts), the log_2_ fold change (log2FC), the standard error of the log_2_ fold change estimate (lfcSE), the Wald statistic (stat), the *p*-value, and the *p*-value adjusted for multiple testing (adjusted *p*-value). Genes with higher expression in medium are colored in blue, and genes with higher expression in infected leaf tissue are colored in green. (XLS 80 kb) Additional file 9:
**Table S6.** Short, cysteine-rich, secreted proteins differentially expressed in infected leaf tissue versus mycelium grown in medium. SignalP 4.1 was used to predict proteins that are secreted from the cell. Proteins predicted to be short (<300 amino acid residues for the mature sequence), cysteine-rich (>4 cysteines), secreted, and for which the corresponding transcript is differentially expressed are shown in the table. For each protein sequence, the table indicates JGI protein and gene IDs, the location of the gene within the genome (including scaffold number, start and stop positions), the log_2_ fold change, the adjusted *p*-value for differential expression of the corresponding transcript, the length of the mature protein in amino acid residues, the number of cysteines in the mature sequence, and blastp results from a search against the NCBI non-redundant protein sequence database. Blastp results include the species where the closest homolog for the sequence was found, the description of the homolog, its NCBI accession number, the bitscore, the E-value, percent identity and similarity, notes on other species where homologs were found, and any conserved domains identified using the Conserved Domain Database. Protein sequences for which the corresponding transcripts have higher expression in infected leaf tissue are colored green, and those with higher expression in medium are colored blue. (XLS 64 kb) Additional file 10:
**Table S7.** Blastp results for genes in differentially expressed gene clusters in infected leaf tissue. For each gene in differentially expressed gene clusters, tables indicate its description in Figs. 7 and 9, and Additional file 13: Figures S7 and Additional file 14: Figure S8, the JGI gene and protein IDs, the log_2_ fold change, the adjusted *p*-value for differential expression, the NCBI accession number, the location of the gene on the *M. fijiensis* genome scaffold, GO, KOG, and Interpro descriptions from JGI, domains identified using the Conserved Domain Database, and the top 10 blastp hits. Blastp hit information includes a description of the hit, the species where the hit was found, bitscore, E-value, percent identity and similarity, and the NCBI accession. Each tab within the Excel file describes a different cluster, with tabs A-M showing clusters with higher expression in infected leaf tissue (colored in green), and tabs N-R showing clusters with higher expression in culture medium (colored in blue). (XLS 1644 kb) Additional file 11:
**Table S8.** Fusicoccadiene synthase homologs used for generation of phylogenetic tree. The table includes the *M. fijiensis* fusicoccadiene synthase and its top homologs as identified by blastp, which were used to generate a phylogenetic tree in Additional file 12: Figure S4. For each sequence, the species where it is found, its description, the bitscore, the E-value, the NCBI accession number, and percent identity with the *M. fijiensis* sequence are indicated. (XLS 66 kb) Additional file 12:
**Figure S4.** Phylogenetic tree of fusicoccadiene synthase protein sequences. A maximum likelihood tree was created of the *M. fijiensis* fusicoccadiene synthase sequence and its top 50 hits using blastp with the non-redundant protein sequence database on NCBI. Bootstrap values are indicated on the tree, and the scale bar of branch lengths indicate substitutions per site. A description of each blast hit is shown, along with an abbreviation for species. *Ab* = *Alternaria brassicicola*; *Ac* = *Acremonium chrysogenum*; *Af* = *Aspergillus flavus*; *Ak* = *Aspergillus kawachii*; *An* = *Aspergillus niger*; *Ao* = *Aspergillus oryzae*; *Ar* = *Aspergillus ruber*; *Bm* = *Bipolaris maydis*; *Bo* = *Bipolaris oryzae*; *Bp* = *Baudoinia panamericana*; *Bs* = *Bipolaris sorokiniana*; *Bv* = *Bipolaris victoriae*; *Bz* = *Bipolaris zeicola*; *Cg* = *Chaetomium globosum*; *Ci* = *Coccidioides immitis*; *Cp* = *Coccidioides posadasii*; *Da* = *Diaporthe amygdali*; *Fg* = *Fusarium graminearum*; *Fp* = *Fusarium pseudograminearum*; *Gl* = *Gymnopus luxurians*; *Mo* = *Magnaporthe oryzae*; *Mp* = *Macrophomina phaseolina*; *Nf* = *Neosartorya fischeri*; *Nu* = *Neosartorya udagawae*; *Om* = *Oidiodendron maius*; *Pp* = *Pseudogymnoascus pannorum*; *Ptt* = *Pyrenophora teres f. teres*; *Tc* = *Talaromyces cellulolyticus*; *Ti* = *Talaromyces islandicus*; *Tm* = *Talaromyces marneffei*; *To* = *Tolypocladium ophioglossoides*; *Ts* = *Talaromyces stipitatus*; *Tt* = *Thielavia terrestris*. (TIF 303 kb) Additional file 13:
**Figure S5.** Gene clusters up-regulated in infected leaf tissue compared to liquid medium. The description of each gene as determined by blastp of the corresponding protein is shown along with its log_2_ fold change of expression in infected leaf tissue versus expression in liquid medium. Black bars are proportional to the log_2_ fold change. Gene expression differences that are significant at *p* < 0.01 are shown with two asterisks above the corresponding bar, and those significant at *p* < 0.05 are shown with a single asterisk. NE = no expression detected. Genes in the putative gene cluster are indicated by blue boxes, and genes flanking the cluster are indicated by gray boxes. A) Gene cluster on scaffold 7; B) Gene cluster on scaffold 21; C) Gene cluster on scaffold 6; D) Gene cluster on scaffold 10; E) Gene cluster on scaffold 1; F) Gene cluster on scaffold 6. (TIF 409 kb) Additional file 14:
**Figure S6.** Gene clusters down-regulated in infected leaf tissue compared to liquid medium. The description of each gene as determined by blastp of the corresponding protein is shown along with its log_2_ fold change value of expression in infected leaf tissue versus expression in liquid medium. Black bars are proportional to the log_2_ fold change value. Gene expression differences that are significant at *p* < 0.01 are shown with two asterisks above the corresponding bar, and those significant at *p* < 0.05 are shown with a single asterisk. NE = no expression detected. Genes in the putative gene cluster are indicated by blue boxes, and genes flanking the cluster are indicated by gray boxes. A) Gene cluster on scaffold 4; B) Gene cluster on scaffold 7; C) Gene cluster on scaffold 8; D) Gene cluster on scaffold 3; E) Gene cluster on scaffold 2. (TIF 541 kb) Additional file 15:
**Table S9.** Homologs of genes on scaffolds 15 and 21 of the *M. fijiensis* genome. For each gene on scaffolds 15 and 21, a blastp search was done using the NCBI non-redundant protein sequence database to identify conserved domains and the best 10 homologs by bitscore for each sequence. For each *M. fijiensis* sequence of interest, the table includes the JGI protein and gene IDs, a link to the NCBI accession, the location of the gene on each scaffold and a link to the genome browser, the log_2_ fold change (log2FC), the *p*-value for differential expression adjusted for multiple testing (adjusted *p*-value), GO, Interpro, and KOG annotations from JGI, conserved domains identified using NCBI’s Conserved Domain Database, and a summary of blastp results using the NCBI nr protein sequence database: species where the homolog was found, a description of the homolog, the bitscore, the E-value, the percent identity and similarity, and a link to the NCBI accession. Genes with higher expression in infected leaf tissue are colored in green. (XLS 235 kb) Additional file 16:
**Figure S7.** Homologs of genes on scaffolds 15 and 21 of the *M. fijiensis* genome. For each gene on scaffolds 15 and 21, a blastp search was done using the NCBI non-redundant protein sequence database to identify homologs and conserved domains. The charts indicate the percent of genes on the scaffolds with: no homologs or conserved domains identified (dark blue), homologs present in species within Mycosphaerellaceae (purple), homologs present in other fungi but not in non-fungal organisms (yellow), and homologs present in non-fungal organisms (light blue). (TIF 262 kb) Additional file 17:
**Figure S8.** Translated nucleotide sequence alignment of Scaffold 20 hypothetical gene in CIRAD86 and 14H1-11A isolates. Chimera 1.10.2 was used to display the degree of conservation for each residue within the alignment of translated nucleotide sequences from the scaffold 20 hypothetical gene from isolates CIRAD86 and 14H1-11A. Amino acid residues corresponding to translated nucleotide sequences are color coded according to the ClustalX [111] color scheme. Asterisks indicate stop codons in the 14H1-11A sequence. Clustal histogram bars are used to indicate sequence conservation, so that larger histogram bars correspond to amino acid residues with more similar physio-chemical properties. (TIF 307 kb) Additional file 18:
**Table S10.** RT-qPCR primers used for validation of RNA-Seq results. For each gene of interest, the first tab of the Excel spreadsheet shows the name of the gene, the name and sequence of the forward and reverse primers, and the expected size of the product. The second tab of the spreadsheet shows the reference gene used in Fig. 1 for each gene of interest. (XLS 9 kb) Additional file 19:
**Table S11.** PCR amplification conditions for scaffold 13 and 20 genes. For each gene amplified from scaffolds 13 and 20, the table indicates the gene’s description in Fig. 11, its NCBI accession number, the JGI Gene ID, the scaffold from which it is amplified, the description of the protein product, the sequences of the forward and reverse primers used for amplification, the annealing temperature, the extension time, and the expected product size. (XLS 12 kb)

## Abbreviations

ABC: ATP-binding cassette
BAP: 6-benzylaminopurine
CAZy: Carbohydrate-active enzymes
CFEM: Common in fungal extracellular membrane
DUF3328: Domain of unknown function 3328
FDR: False discovery rate
GO: Gene ontology
HsbA: Hydrophobic surface binding protein A
JGI: Joint Genome Institute
log2FC: log_2_ fold change
MFS: Major facilitator superfamily
NCBI: National Center for Biotechnology Information
NRPS: Non-ribosomal peptide synthase
PBSA: Polybutylene succinate-co-adipate
PDB: Potato dextrose broth
PR-1: Pathogenesis-related protein 1
