# Supplementary figures and images for: Transcriptome sequencing of Mycosphaerella fijiensis during association with Musa acuminata reveals candidate pathogenicity genes
Source: BMC Genomics. 2016 Aug 30;17(1):690. doi: 10.1186/s12864-016-3031-5 (PMC5006380; doi:10.1186/s12864-016-3031-5)

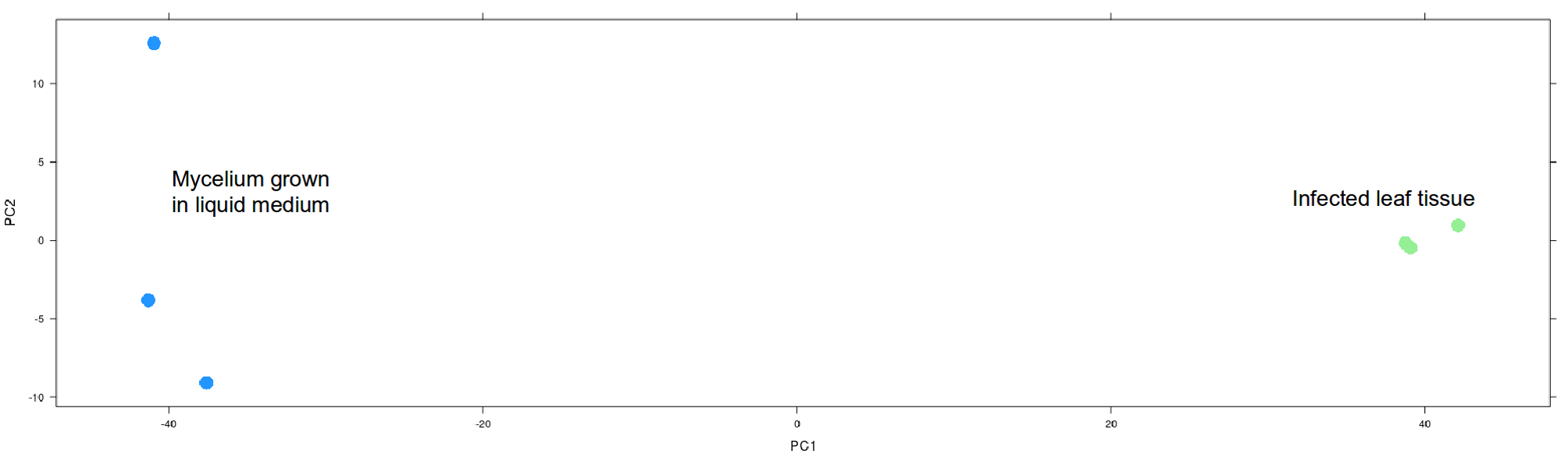

Supplement: Additional file 1: — Figure S1. Two-dimensional principal component analysis of infected leaf samples versus mycelium grown in liquid culture. Infected leaf samples are shown as green dots, and samples of mycelium grown in PDB medium are shown as blue dots. (TIF 67 kb) [file 12864_2016_3031_MOESM1_ESM.tif]

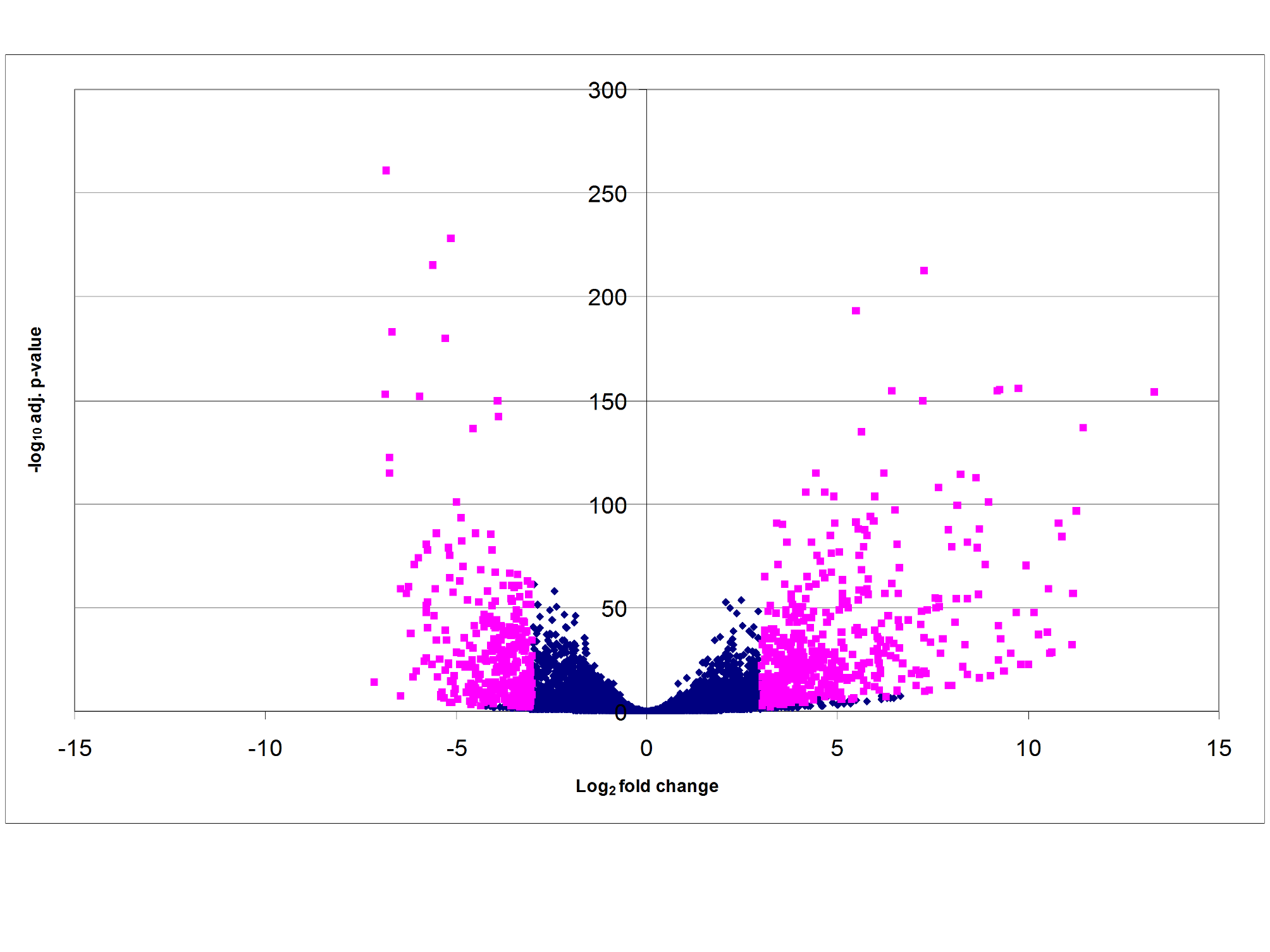

Supplement: Additional file 2: — Figure S2. Volcano plot showing the distribution of differentially expressed sequences. The horizontal axis shows the log2 fold change for expression in infected leaf tissue versus the fungus grown in liquid medium, and the vertical axis shows the -log10 (p-value). Each transcript is represented by a dot, which is colored pink for each of the differentially expressed transcripts listed in Additional file 4: Table S2, and colored blue for transcripts that are not differentially expressed. (TIF 154 kb) [file 12864_2016_3031_MOESM2_ESM.tif]

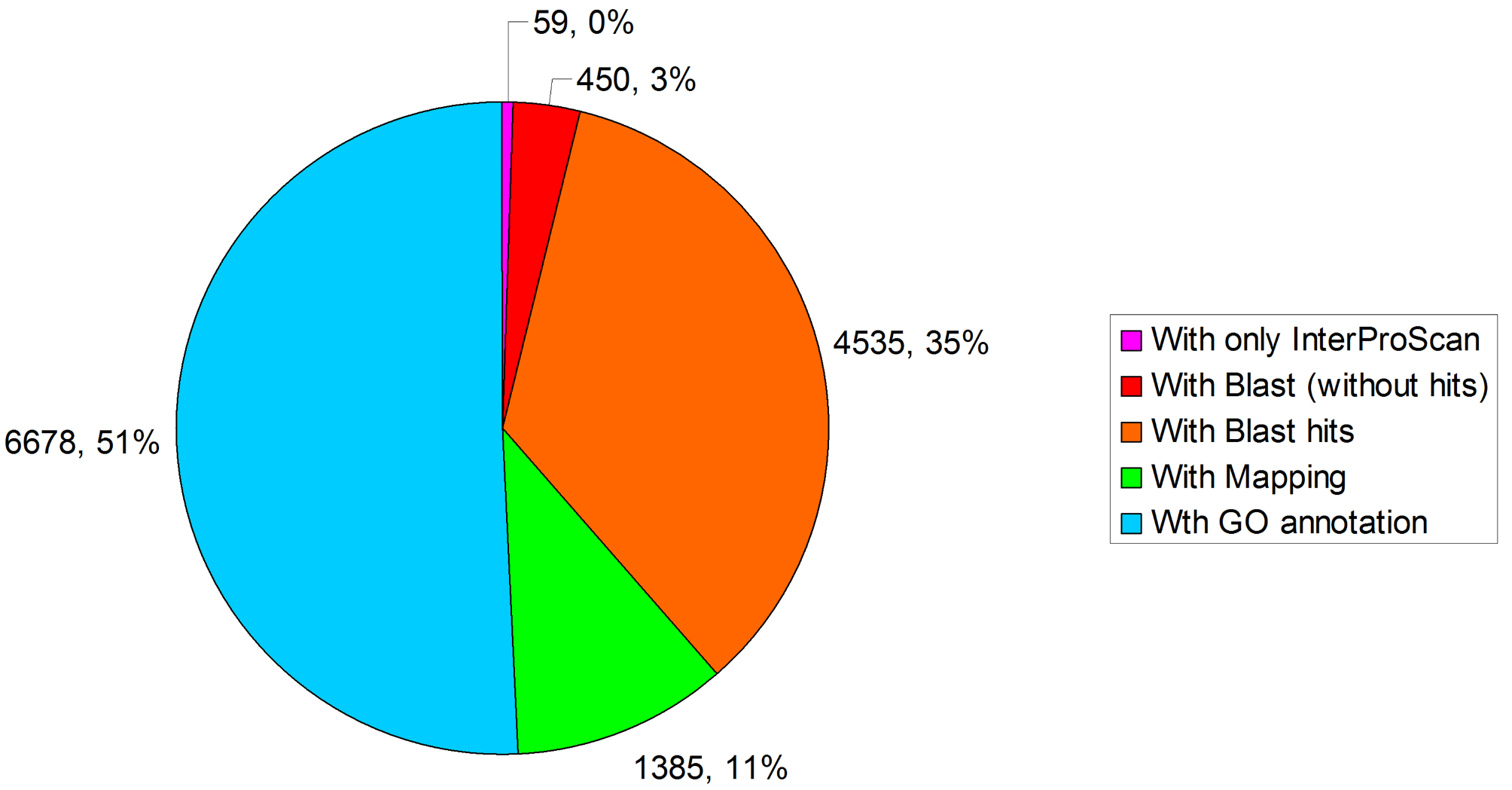

Supplement: Additional file 7: — Figure S3. Annotation results of all predicted genes in the M. fijiensis genome, using Blast2GO. Red = Sequences for which blast analysis was done, but no hits were found. Orange = Sequences for which blast analysis was done and hits were found, but no GO terms were mapped to the hits. Green = Sequences for which blast analysis was done and GO terms were successfully mapped to the hits. Blue = Query sequences to which GO terms were successfully annotated. Pink = Sequences for which only the InterProScan results were obtained. (TIF 256 kb) [file 12864_2016_3031_MOESM7_ESM.tif]

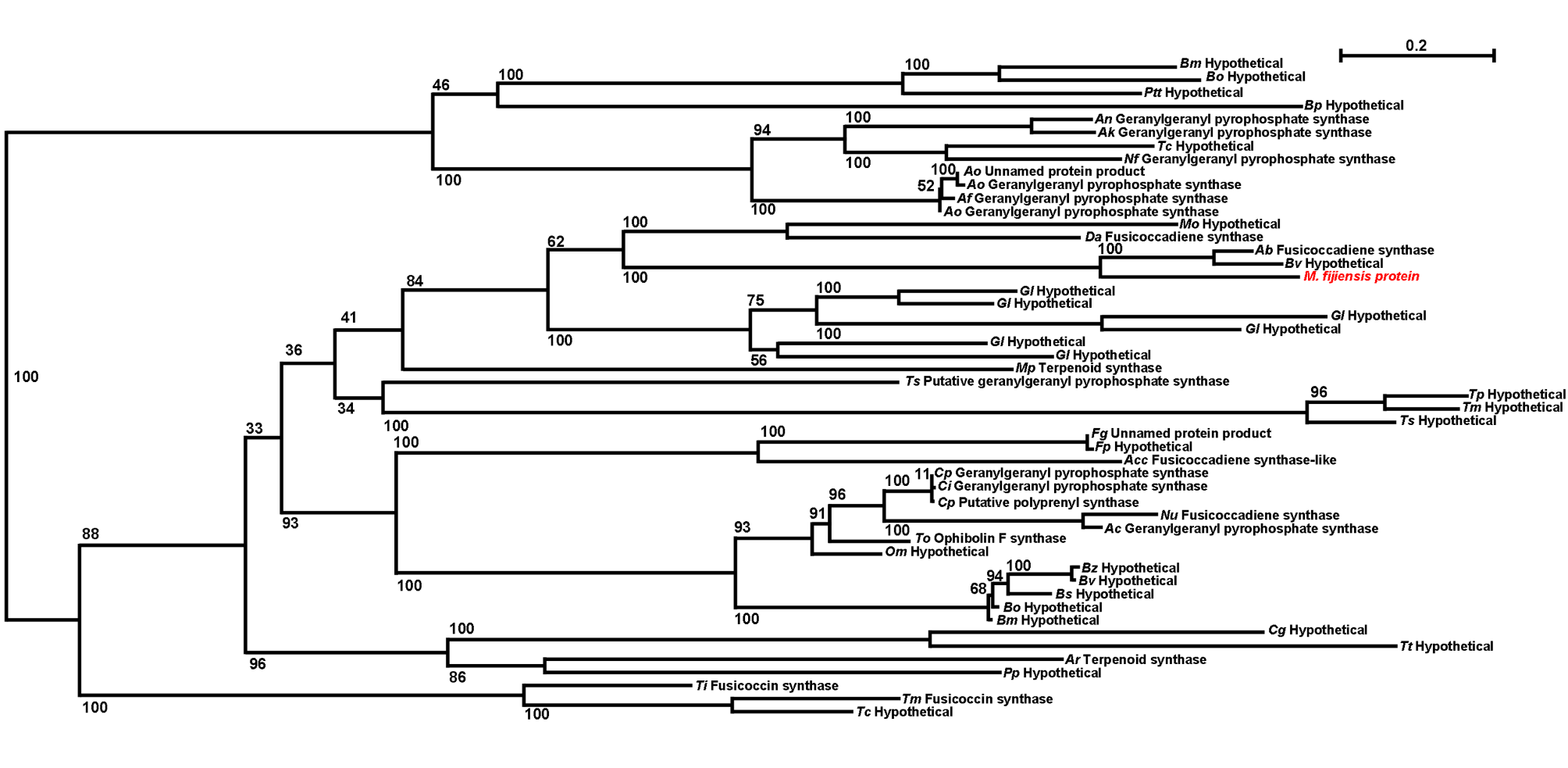

Supplement: Additional file 12: — Figure S4. Phylogenetic tree of fusicoccadiene synthase protein sequences. A maximum likelihood tree was created of the M. fijiensis fusicoccadiene synthase sequence and its top 50 hits using blastp with the non-redundant protein sequence database on NCBI. Bootstrap values are indicated on the tree, and the scale bar of branch lengths indicate substitutions per site. A description of each blast hit is shown, along with an abbreviation for species. Ab = Alternaria brassicicola; Ac = Acremonium chrysogenum; Af = Aspergillus flavus; Ak = Aspergillus kawachii; An = Aspergillus niger; Ao = Aspergillus oryzae; Ar = Aspergillus ruber; Bm = Bipolaris maydis; Bo = Bipolaris oryzae; Bp = Baudoinia panamericana; Bs = Bipolaris sorokiniana; Bv = Bipolaris victoriae; Bz = Bipolaris zeicola; Cg = Chaetomium globosum; Ci = Coccidioides immitis; Cp = Coccidioides posadasii; Da = Diaporthe amygdali; Fg = Fusarium graminearum; Fp = Fusarium pseudograminearum; Gl = Gymnopus luxurians; Mo = Magnaporthe oryzae; Mp = Macrophomina phaseolina; Nf = Neosartorya fischeri; Nu = Neosartorya udagawae; Om = Oidiodendron maius; Pp = Pseudogymnoascus pannorum; Ptt = Pyrenophora teres f. teres; Tc = Talaromyces cellulolyticus; Ti = Talaromyces islandicus; Tm = Talaromyces marneffei; To = Tolypocladium ophioglossoides; Ts = Talaromyces stipitatus; Tt = Thielavia terrestris. (TIF 303 kb) [file 12864_2016_3031_MOESM12_ESM.tif]

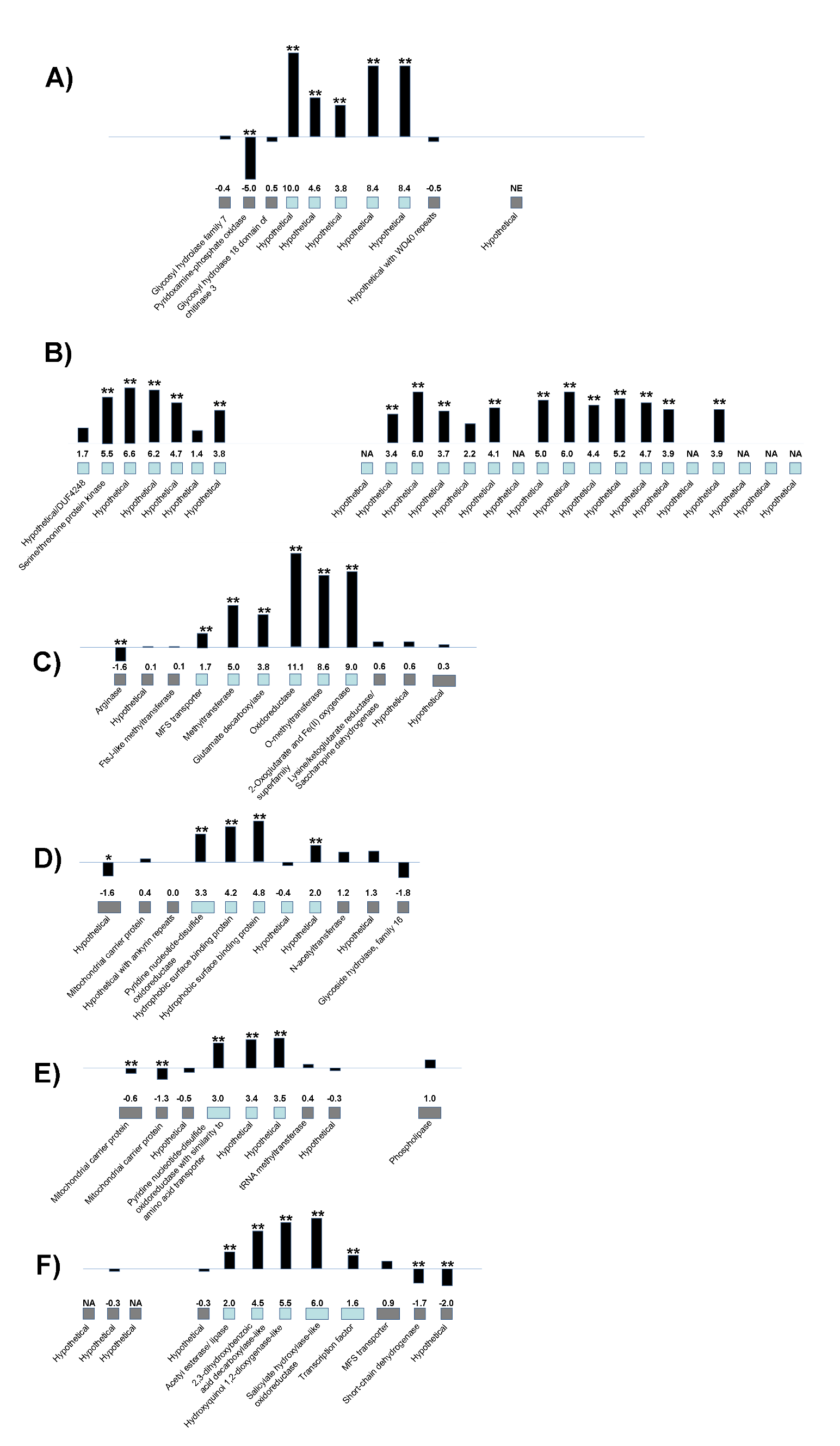

Supplement: Additional file 13: — Figure S5. Gene clusters up-regulated in infected leaf tissue compared to liquid medium. The description of each gene as determined by blastp of the corresponding protein is shown along with its log2 fold change of expression in infected leaf tissue versus expression in liquid medium. Black bars are proportional to the log2 fold change. Gene expression differences that are significant at p < 0.01 are shown with two asterisks above the corresponding bar, and those significant at p < 0.05 are shown with a single asterisk. NE = no expression detected. Genes in the putative gene cluster are indicated by blue boxes, and genes flanking the cluster are indicated by gray boxes. A) Gene cluster on scaffold 7; B) Gene cluster on scaffold 21; C) Gene cluster on scaffold 6; D) Gene cluster on scaffold 10; E) Gene cluster on scaffold 1; F) Gene cluster on scaffold 6. (TIF 409 kb) [file 12864_2016_3031_MOESM13_ESM.tif]

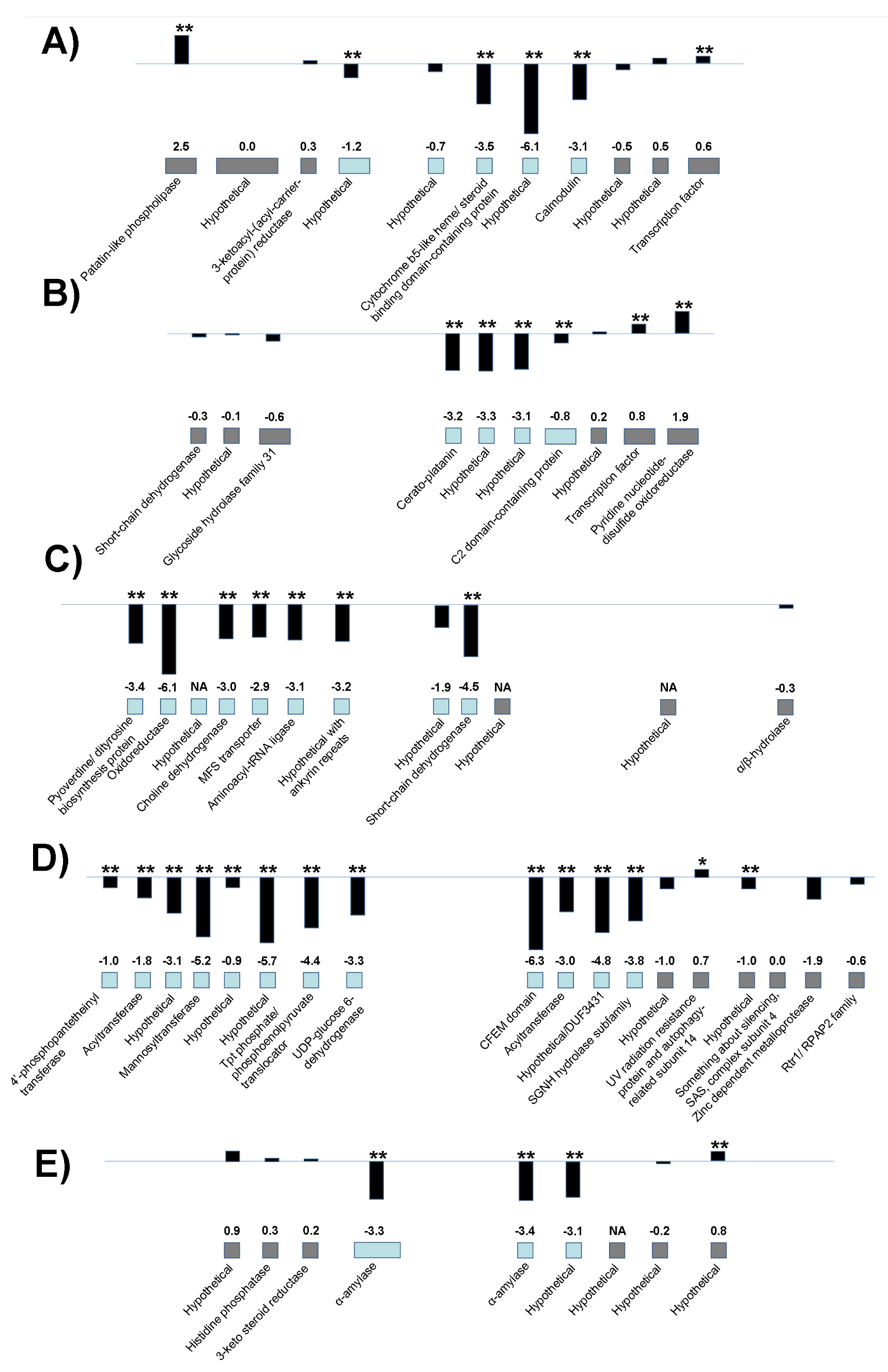

Supplement: Additional file 14: — Figure S6. Gene clusters down-regulated in infected leaf tissue compared to liquid medium. The description of each gene as determined by blastp of the corresponding protein is shown along with its log2 fold change value of expression in infected leaf tissue versus expression in liquid medium. Black bars are proportional to the log2 fold change value. Gene expression differences that are significant at p < 0.01 are shown with two asterisks above the corresponding bar, and those significant at p < 0.05 are shown with a single asterisk. NE = no expression detected. Genes in the putative gene cluster are indicated by blue boxes, and genes flanking the cluster are indicated by gray boxes. A) Gene cluster on scaffold 4; B) Gene cluster on scaffold 7; C) Gene cluster on scaffold 8; D) Gene cluster on scaffold 3; E) Gene cluster on scaffold 2. (TIF 541 kb) [file 12864_2016_3031_MOESM14_ESM.tif]

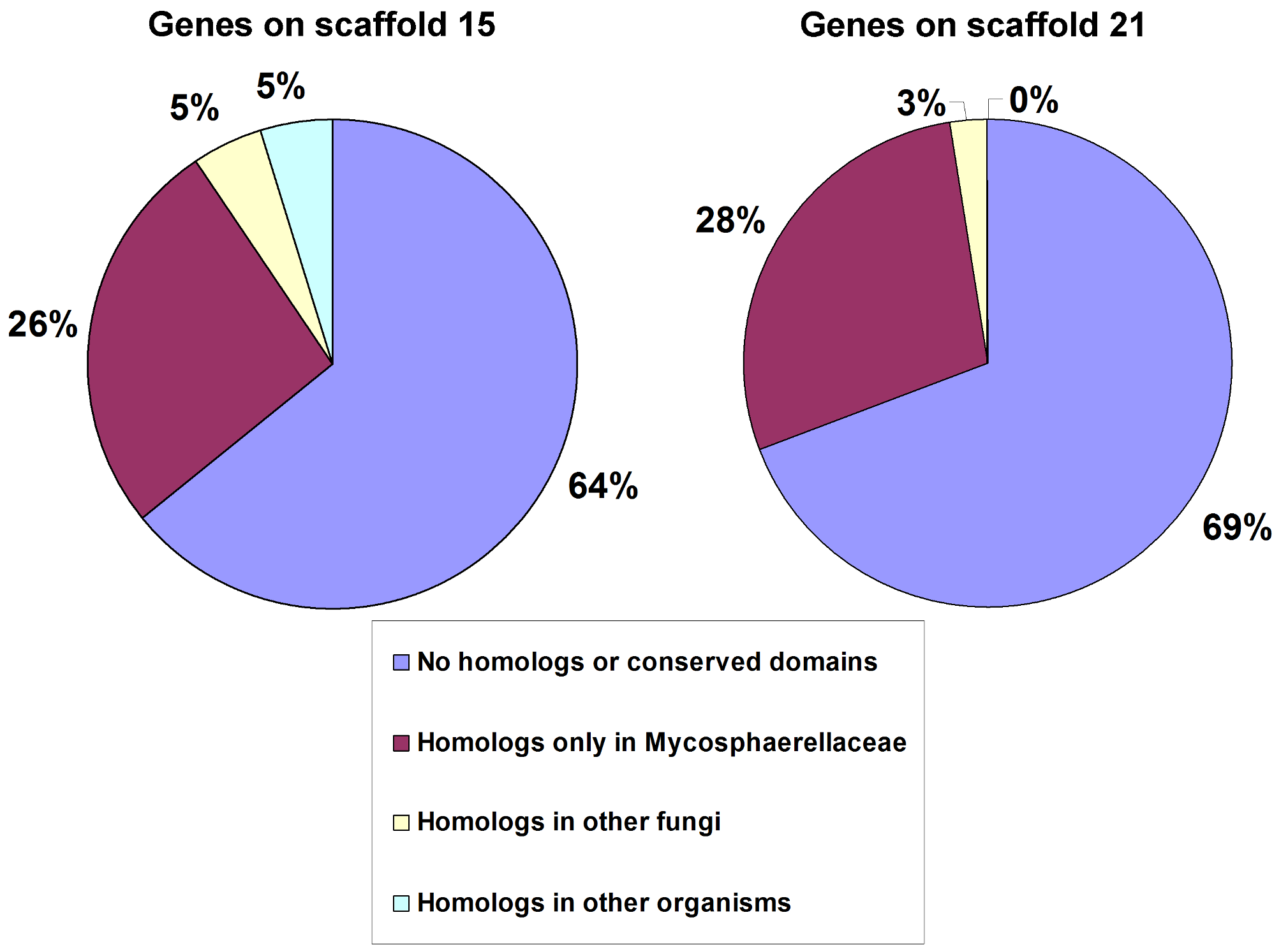

Supplement: Additional file 16: — Figure S7. Homologs of genes on scaffolds 15 and 21 of the M. fijiensis genome. For each gene on scaffolds 15 and 21, a blastp search was done using the NCBI non-redundant protein sequence database to identify homologs and conserved domains. The charts indicate the percent of genes on the scaffolds with: no homologs or conserved domains identified (dark blue), homologs present in species within Mycosphaerellaceae (purple), homologs present in other fungi but not in non-fungal organisms (yellow), and homologs present in non-fungal organisms (light blue). (TIF 262 kb) [file 12864_2016_3031_MOESM16_ESM.tif]

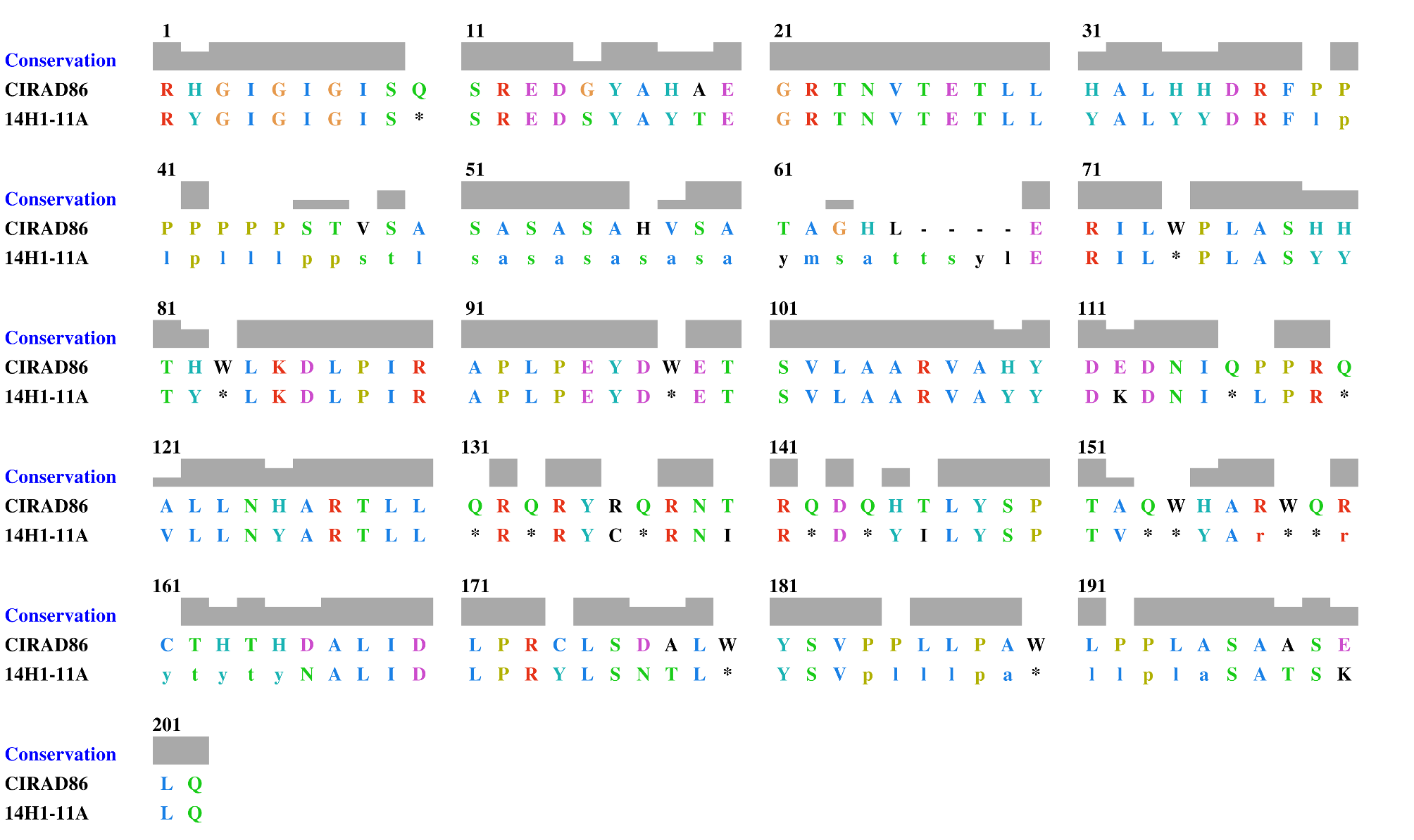

Supplement: Additional file 17: — Figure S8. Translated nucleotide sequence alignment of Scaffold 20 hypothetical gene in CIRAD86 and 14H1-11A isolates. Chimera 1.10.2 was used to display the degree of conservation for each residue within the alignment of translated nucleotide sequences from the scaffold 20 hypothetical gene from isolates CIRAD86 and 14H1-11A. Amino acid residues corresponding to translated nucleotide sequences are color coded according to the ClustalX [111] color scheme. Asterisks indicate stop codons in the 14H1-11A sequence. Clustal histogram bars are used to indicate sequence conservation, so that larger histogram bars correspond to amino acid residues with more similar physio-chemical properties. (TIF 307 kb) [file 12864_2016_3031_MOESM17_ESM.tif]
